# FTO-mediated demethylation of PRKCB mRNA activates NF-κB to trigger alveolar fibrin deposition in ARDS

**DOI:** 10.3389/fimmu.2026.1960313

**Published:** 2026-10-14

**Authors:** Xianjun Chen, Ying Liu, Song Mu, Tingting Chen, Jia Yuan, Chuan Xiao, Qi Dong, Qing Li, Ying Wang, Qimin Chen, Shuwen Li, Wei Li, Feng Shen

**Affiliations:** 1 Department of Critical Care Medicine, The Affiliated Hospital of Guizhou Medical University, Guiyang, China; 2 Department of Respiratory and Critical Care Medicine, Wuhan No. 1 Hospital, Wuhan, China; 3 Department of Anorectal Surgery, The Affiliated Hospital of Guizhou Medical University, Guiyang, China; 4 Houshan Township Health Center, Zunyi, China

**Keywords:** acute respiratory distress syndrome (ARDS), alveolar fibrin deposition, FTO alpha-ketoglutarate dependent dioxygenase (FTO), N^6^-methylAdenosine (m^6^A), NF-κB signaling, protein kinase C beta (PRKCB)

## Abstract

**Background:**

Acute Respiratory Distress Syndrome (ARDS) is characterized by alveolar fibrin deposition, driven by dysregulated hypercoagulation and impaired fibrinolysis. Although alveolar epithelial type II (AEC II) cells contribute to this imbalance by overexpressing Tissue Factor (TF) and Plasminogen Activator Inhibitor-1 (PAI-1), the upstream regulatory mechanisms, particularly at the epitranscriptomic level, remain unclear. The potential role of the N6-methyladenosine (m^6^A) RNA demethylase FTO in ARDS pathogenesis is unexplored.

**Methods:**

In this study, we employed lipopolysaccharide (LPS)-induced rat ARDS models and LPS-stimulated AEC II cells as primary experimental systems to investigate the underlying mechanisms. Multi-omics profiling, combining methylated RNA immunoprecipitation sequencing (MeRIP-seq) and RNA sequencing (RNA-seq), was performed. Gain and loss-of-function studies were conducted using lentiviral vectors both *in vitro* and *in vivo*. Mechanistic investigations involved MeRIP-qPCR, mRNA stability assays, co-immunoprecipitation (Co-IP), Western blot, immunofluorescence, and pharmacological modulation of the NF-κB pathway.

**Results:**

Lungs from ARDS rats showed decreased global levels and increased FTO expression. Knockdown of FTO alleviated lung injury, reduced pulmonary edema, and suppressed the expression of TF and PAI-1, concomitant with inhibition of NF-κB signaling. Integrated multi-omics analysis identified Protein Kinase C Beta (PRKCB), a serine/threonine kinase known to regulate inflammatory signaling, as a key target of FTO. Mechanistically, FTO-mediated demethylation of PRKCB mRNA reduced its recognition by reader proteins that typically promote mRNA decay, thereby prolonging its half-life and enhancing protein translation. Elevated PRKCB promoted IKKβ phosphorylation and subsequent NF-κB activation. Rescue experiments confirmed that FTO regulates alveolar hypercoagulation and fibrinolytic inhibition in ARDS via PRKCB-dependent activation of the NF-κB pathway.

**Conclusion:**

Our study identifies the FTO-PRKCB-NF-κB axis as a novel epitranscriptomic pathway driving alveolar fibrin deposition in ARDS. These findings reveal FTO as a potential therapeutic target for mitigating alveolar hypercoagulation and fibrinolysis inhibition in ARDS.

## Graphical Abstract

**Figure:**
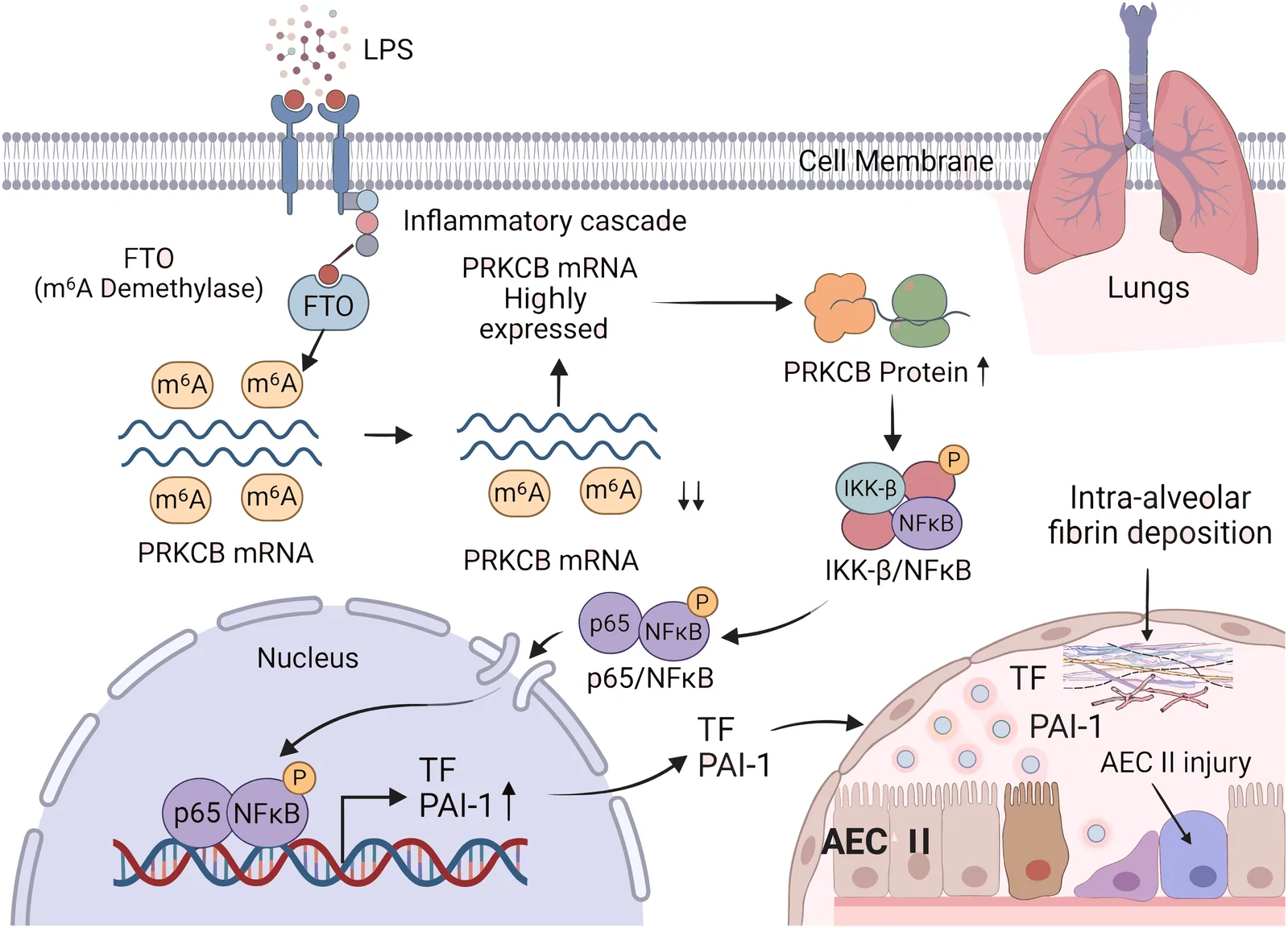
The FTO-PRKCB-NF-κB axis in ARDS. LPS-induced inflammation upregulates FTO, which removes m⁶A methylation from PRKCB mRNA, increasing PRKCB stability and expression. PRKCB then promotes IKKβ/NF-κB activation, driving TF and PAI-1 expression and extracellular alveolar fibrin deposition. FTO represents a potential therapeutic target for ARDS-associated coagulopathy.

## 1 Introduction

Acute Respiratory Distress Syndrome (ARDS), a leading cause of respiratory failure in intensive care units (ICUs) (1), is associated with persistently high mortality rates (2). A hallmark pathological feature of ARDS is intra-alveolar fibrin deposition, which severely compromises gas exchange (3, 4). This phenomenon arises from a pronounced local imbalance between coagulation and fibrinolysis (5, 6), characterized by enhanced tissue factor (TF)-mediated procoagulant activity and impaired fibrinolytic capacity due to elevated levels of plasminogen activator inhibitor-1 (PAI-1) (7–10). Therefore, restoring balance in alveolar hemostasis represents a critical therapeutic objective.

Alveolar epithelial type II cells (AEC IIs) are now recognized as active participants in this dysregulation, capable of upregulating both TF and PAI-1 in response to injury stimuli such as lipopolysaccharide (LPS) (11, 12). The NF-κB signaling pathway serves as a central regulator of the procoagulant and inflammatory responses in injured AEC IIs (13). However, the upstream regulatory signals and, more importantly, the post-transcriptional mechanisms governing this response in the context of ARDS remain poorly defined. While neutrophil-dominated alveolar inflammation is a well-recognized hallmark of ARDS (14), increasing evidence indicates that dysregulated coagulation and fibrinolysis within the alveolar space-leading to fibrin deposition-constitute an equally critical but less therapeutically addressed pathological feature.

Emerging evidence highlights epitranscriptomic regulation, particularly reversible N6-methyladenosine (m^6^A) modification of mRNA, as a pivotal layer of gene expression control (15). The demethylase fat mass and obesity-associated protein (FTO), which has been implicated in various diseases, including inflammatory disorders, has been shown to interact with the NF-κB pathway (16–18). Nonetheless, the functional role of FTO and -mediated RNA regulation in alveolar epithelial injury and the associated coagulopathy in ARDS remains unexplored.

We hypothesized that FTO functions as an upstream epitranscriptomic regulator that promotes procoagulant and antifibrinolytic responses in AEC IIs during ARDS through demethylation and subsequent activation of the NF-κB pathway. In this study, we observed upregulated FTO expression accompanied by a global reduction in methylation in experimental models of ARDS. Integrated methylated RNA immunoprecipitation sequencing (MeRIP-seq) and RNA sequencing (RNA-seq) analyses identified Protein Kinase C Beta (PRKCB) mRNA as a key downstream target of FTO. We demonstrate that FTO-mediated demethylation enhances PRKCB mRNA stability, leading to increased PRKCB protein expression. This, in turn, activates the IKK-NF-κB signaling axis, resulting in the transcriptional upregulation of TF and PAI-1. Genetic rescue experiments further validated a linear causal pathway FTO-PRKCB-NF-κB that drives alveolar hypercoagulability and suppression of fibrinolysis.

Notably, a recent study demonstrated that FTO-mediated demethylation of CRTC1 coordinates with p300 and EGR2 to promote alveolar extracellular matrix degradation in ARDS (19), further underscoring the broad regulatory roles of FTO in alveolar injury. However, whether FTO regulates alveolar coagulation and fibrinolytic balance, a distinct but complementary aspect of ARDS pathophysiology, remains unknown. This study identifies a previously unrecognized epitranscriptomic regulatory axis, the FTO-PRKCB-NF-κB pathway, that plays a critical role in mediating alveolar fibrin accumulation in ARDS. Our findings provide mechanistic insights into the molecular regulation of alveolar coagulopathy and position FTO as a promising therapeutic target for mitigating lung injury in this severe clinical syndrome.

## 2 Methods

### 2.1 Animal studies

All procedures were approved by the Animal Research Ethics Committee of Guizhou Medical University (Approval No. 2503330) and conducted in accordance with the ARRIVE guidelines. To minimize confounding effects of sex hormones on inflammatory and coagulation responses, only male rats were used in this study, consistent with previous ARDS models (20). Each experimental group consisted of 6 rats (n=6 per group), determined by power analysis based on preliminary data. Male Sprague-Dawley rats (6–8 weeks, 220 ± 25 g, Beijing Vital River Laboratory Animal Technology Co., Ltd. Certificate No. 110011231106283975) were maintained under specific pathogen-free conditions. For ARDS induction, rats were anesthetized with 4% isoflurane (RWD Life Science, China) via inhalation. After adequate anesthesia, rats were secured in a supine position, the glottis was exposed using a laryngoscope, and a microsprayer (Beijing Yuansen Biotechnology, China) was inserted approximately 0.5 cm below the vocal cords. Lipopolysaccharide (LPS, Sigma-Aldrich, USA, Cat# L2880) was dissolved in sterile saline at 5 mg/mL and administered via airway nebulization at 10 mg/kg (20). Control rats received an equal volume of sterile saline. At 24 h after LPS administration, rats were anesthetized with 4% isoflurane via inhalation using a small-animal anesthesia machine (RWD Life Science, China) and then euthanized by exsanguination via the abdominal aorta. Lung tissues were subsequently harvested for further analyses. For lentivirus delivery, rats were anesthetized with 4% isoflurane and secured in a supine position. A laryngoscope was used to expose the glottis, and a microsprayer (Beijing YSKD) was inserted approximately 0.5 cm below the vocal cords. Lentiviral particles (1 × 10^8^ TU in 250 μL) were aerosolized intratracheally to ensure uniform pulmonary distribution (21). At 24 h after LPS administration, rats were anesthetized with 4% isoflurane via inhalation and euthanized by exsanguination via the abdominal aorta, as described above. All lentiviral procedures were performed in a biosafety cabinet to minimize aerosol exposure, and the nebulizer was handled according to institutional biosafety guidelines.

#### 2.1.1 Tissue collection

Lung tissues were harvested forbronchoalveolar lavage fluid (BALF) collection, wet/dry weight ratio measurement, histopathology (fixed in 4% paraformaldehyde), and snap-freezing in liquid nitrogen for protein/RNA extraction. BALF was collected by instilling 3 mL of pre-cooled sterile saline into the left upper bronchus (three washes; recovery rate 60-65%) and centrifuged at 1,200 × g for 10 min at 4 °C; supernatants were stored at -80 °C.

#### 2.1.2 Lung injury assessment

Histopathological evaluation was performed on paraffin-embedded sections (4μm) stained with hematoxylin and eosin. Lung injury was scored using the diffuse alveolar damage (DAD) system as described by Matute-Bello et al (22). The calculation method is shown in **Supplementary Table 1**. Collagen III expression was detected by immunohistochemistry (anti-Collagen III, 1:100, Servicebio, Cat# GB111629) with DAB development (Beyotime, Cat# P0203) and scored based on positive cell percentage and staining intensity. Wet/dry weight ratio was calculated after drying at 60 °C until constant weight.

#### 2.1.3 ELISA

Concentrations of TF, PAI-1, and thrombin in BALF were measured using ELISA kits (TF: Jingmei, Cat# JM-02321R1; PAI-1: Jingmei, Cat# JM-02322R1; thrombin: Mian Biotechnology, Cat# MB-7235A) according to the manufacturers’ instructions. Absorbance was read at 450 nm.

#### 2.1.4 Global quantification

Global levels in lung tissues were quantified using the RNA Methylation Quantification Kit (Colorimetric, Amyjet, Cat# AMJS-0009). Briefly, 200 ng of RNA was incubated with capture and detection antibodies, followed by colorimetric development, and absorbance was measured at 450 nm. amount was calculated from a standard curve.

### 2.2 Cell culture and treatments

The rat alveolar epithelial type II cell line RLE-6TN (Xiangya Cell Center) was cultured in RPMI-1640 (Gibco, Cat# 11875093) supplemented with 10% FBS (Gibco, Cat# 10270106) and 1% penicillin-streptomycin (Gibco, Cat# 15140122) at 37 °C in 5% CO_2_. Cells were stimulated with LPS (5 μg/mL, Sigma-Aldrich, Cat# L2880) for 24 h. For pathway inhibition, cells were pretreated with the NF-κB inhibitor benzoxathiole (50 μM, Abcam, Cat# ab144574) or the NF-κB agonist HY-134476 (50 μM, EMC, Cat# EMC-0234) for 2 h prior to LPS stimulation. All compounds were dissolved in DMSO (Sigma-Aldrich, Cat# D8418; final concentration ≤ 0.1%).

### 2.3 Lentiviral vector construction and transduction

Lentiviral vectors encoding FTO-specific shRNA (target sequences: sh1, 5′-GCTGCACCTACAAGTACTTGA-3′; sh2, 5′-GCAGCCTACAACGTGACTTTG-3′; sh3, 5′-GCCTGCAGTTCTGAAACAAAGG-3′) or FTO overexpression constructs were packaged by Beijing Tsingke Biotechnology (titer: 1×10^8^ TU/mL). Cells were transduced at a multiplicity of infection (MOI) of 15 for 16 hours, followed by puromycin selection (8μg/mL) for 7 days to establish stable cell lines. For *in vivo* delivery, rats received intratracheal nebulization of lentivirus (1×10^8^ TU in 250μL) 48 hours prior to LPS challenge.

### 2.4 RNA isolation and quantitative real-time PCR

Total RNA was extracted using TRIzol reagent (Invitrogen) according to the manufacturer’s protocol. RNA quality was assessed using a NanoDrop 2000 spectrophotometer (Thermo Fisher Scientific). cDNA was synthesized using the Hifair^®^ III Reverse Transcriptase kit (Yeasen Biotechnology). Quantitative PCR was performed using Hieff UNICON^®^ Universal Blue qPCR SYBR Green Master Mix (Yeasen Biotechnology) on a Bio-Rad CFX96 system. Gene expression was normalized to GAPDH and calculated using the 2^(-ΔΔCt) method. Primer sequences are provided in **Supplementary Table 2**.

### 2.5 Western blot analysis

Total protein was extracted from lung tissues and RLE-6TN cells using RIPA lysis buffer (Beyotime Biotechnology, China) supplemented with protease and phosphatase inhibitors (NCM Biotech, China) and PMSF (Beyotime). Protein concentrations were determined by BCA assay (Beyotime). Equal amounts of protein (30 μg per lane) were separated by 8-12% SDS-PAGE and transferred onto PVDF membranes (Millipore, USA). Membranes were blocked with 5% non-fat milk in TBST for 1 h at room temperature and incubated with primary antibodies overnight at 4 °C, followed by HRP-conjugated secondary antibodies (1:6000, Jackson ImmunoResearch) for 1 h at room temperature. Primary antibodies used were: anti-FTO (1:1000, Abcam), anti-TF (1:2000, NOVUS), anti-PAI-1 (1:500, Proteintech), anti-PRKCB (1:1000, Zen-Bio), anti-phospho-IKKβ (Ser177/181, 1:1000, ABclonal), anti-IKKβ (1:1000, Proteintech), anti-phospho-p65 (Ser536, 1:1000, Cell Signaling Technology), anti-p65 (1:1000, Proteintech), and anti-GAPDH (1:12000, ABclonal). Protein bands were visualized using ECL substrate (Millipore) and quantified with Image J software. Antibody information are provided in **Supplementary Table 3**.

### 2.6 Methylated RNA immunoprecipitation sequencing and RNA-seq

Total RNA was extracted from lung tissues (n = 3 per group) using TRIzol (Invitrogen). Poly(A) mRNA was enriched using oligo(dT) magnetic beads (Thermo Fisher Scientific). For MeRIP-seq, fragmented mRNA was immunoprecipitated with anti- antibody (5 μg, Synaptic Systems) in IP buffer (50 mM Tris-HCl pH 7.4, 750 mM NaCl, 0.5% Igepal CA-630) at 4 °C for 2 h, followed by capture with protein A/G magnetic beads (Thermo Fisher Scientific). Libraries were constructed using the NEBNext Ultra II RNA Library Prep Kit (NEB) and sequenced on an Illumina NovaSeq 6000 platform (PE150). For RNA-seq, libraries were prepared from total RNA using the same kit. Raw reads were processed with fastp and aligned to the Rattus norvegicus reference genome (Ensembl release-101) using HISAT2. peaks were called with exomePeak2 and annotated with ANNOVAR; motif discovery was performed with HOMER. Gene expression levels (FPKM) were quantified using StringTie, and differential expression analysis was performed with edgeR (|log_2_FC| ≥ 1, adjusted p < 0.05). The data are deposited in GEO under accession GSE316047.

### 2.7 MeRIP-seq data analysis

Fastp software was used to remove reads containing adapter sequences, low-quality bases, and undetermined nucleotides using default parameters. The sequence quality of IP and Input samples was assessed with Fast QC. Clean reads were then aligned to the Rattus norvegicus reference genome (http://ftp.ensembl.org/pub/release-101/fasta/rattus_norvegicus/dna/) using HISAT2. Peak calling and differential peak analysis were performed using the R package exomePeak2 (https://github.com/ZW-xjtlu/exomePeak2), and the called peaks were annotated by intersecting with gene architectural features using ANNOVAR. For motif analysis, *de novo* and known motif discovery was conducted using HOMER, followed by mapping the identified motifs relative to the peak summits. Transcript and gene expression levels (FPKM) from the Input libraries were quantified using StringTie. Differential expression analysis was performed with the R package edgeR, selecting transcripts and genes with |log_2_(fold change)| ≥ 1 and an adjusted p-value < 0.05 as statistically significant. The data analysis was assisted by Hangzhou Lianchuan Biological Technology Co., LTD.

### 2.8 MeRIP-qPCR

MeRIP was performed using the EpiQuik CUT&RUN RNA Enrichment Kit (EpiGentek) according to the manufacturer’s instructions. Briefly, 10 μg of total RNA was incubated with antibody or normal mouse IgG overnight at 4 °C. RNA–antibody complexes were captured using protein A/G beads. After washing, bound RNA was eluted and purified for qPCR analysis. The enrichment of target transcripts was calculated as a percentage of input.

### 2.9 mRNA stability assay

RLE-6TN cells were treated with actinomycin D (5 μg/mL, MedChemExpress) to inhibit transcription. Cells were harvested at 0, 0.5, 1, 2, 3 and 4 hours post-treatment. Total RNA was extracted, and PRKCB mRNA levels were determined by qRT-PCR. mRNA half-life was calculated by nonlinear regression analysis using GraphPad Prism 8.0.

### 2.10 Immunofluorescence

Cells grown on coverslips were fixed with 4% paraformaldehyde, permeabilized with 0.3% Triton X-100, and blocked with 5% BSA (Thermo Fisher Scientific). Cells were incubated with primary antibodies against PRKCB (1:100, Zen-Bio) and p-IKKβ (1:100, ABclonal) overnight at 4 °C, followed by Alexa Fluor-conjugated secondary antibodies (1:300, Invitrogen) for 1 h at room temperature. Nuclei were counterstained with DAPI (Beyotime Biotechnology). Images were captured using a Leica TCS SP8 confocal microscope.

### 2.11 Co-immunoprecipitation

To validate the interaction between PRKCB and phosphorylated IKKβ (p-IKKβ), a key activator of the NF-κB signaling pathway, co-immunoprecipitation assays were performed. Briefly, AEC II cells were stimulated with LPS and lysed in 500 μL of RIPA buffer supplemented with protease and phosphatase inhibitors. Cell lysates were divided into three groups: an immunoprecipitation (IP) group, an IgG control group, and an input group. According to the manufacturer’s instructions, the IP group was incubated with an anti-PRKCB antibody, while the IgG control group received 5 μL of normal IgG. Both mixtures were incubated overnight at 4 °C with gentle rotation. Following three washes with phosphate-buffered saline (PBS), 50 μL of Protein A/G magnetic beads were added to each sample and incubated for 4 hours at 4 °C to facilitate antibody-bead conjugation. The beads were then isolated using a magnetic separator, and the supernatant was carefully removed. The bead-bound immune complexes were resuspended in 40 μL of 1× loading buffer and boiled at 100 °C for 10 minutes to elute bound proteins. After a final magnetic separation, the supernatants containing the eluted proteins were collected. Subsequently, samples from the IP, IgG control, and input groups were subjected to western blot analysis to detect p-IKKβ and PRKCB.

### 2.12 Statistical analysis

All data are expressed as mean ± standard deviation (SD) from at least three independent experiments. Statistical analyses were conducted using GraphPad Prism 8.0.2. Differences between two groups were assessed using a two-tailed Student’s t-test, while comparisons among multiple groups were performed using one-way ANOVA followed by Tukey’s *post hoc* test. A *p*-value less than 0.05 was considered statistically significant.

## 3 Results

### 3.1 FTO is upregulated and associated with global reduction of methylation in LPS-induced ARDS

To investigate the role of RNA modification in ARDS, we employed a well-established rat model of LPS-induced ARDS via inhalation (20). LPS challenge (10 mg/kg) induced hallmark pathological features 24 hours post-administration, including severe diffuse alveolar damage, pulmonary edema (**Figures 1A–C**), and increased collagen deposition, as evidenced by positive Masson’s trichrome staining (**Figure 1D**). Concurrently, TF, PAI-1, and thrombin in bronchoalveolar lavage fluid (BALF) were significantly elevated (**Figure 1E**). Moreover, both mRNA and protein expression levels of TF and PAI-1 were upregulated in lung tissues (**Figures 1F–H**).

**Figure 1:**
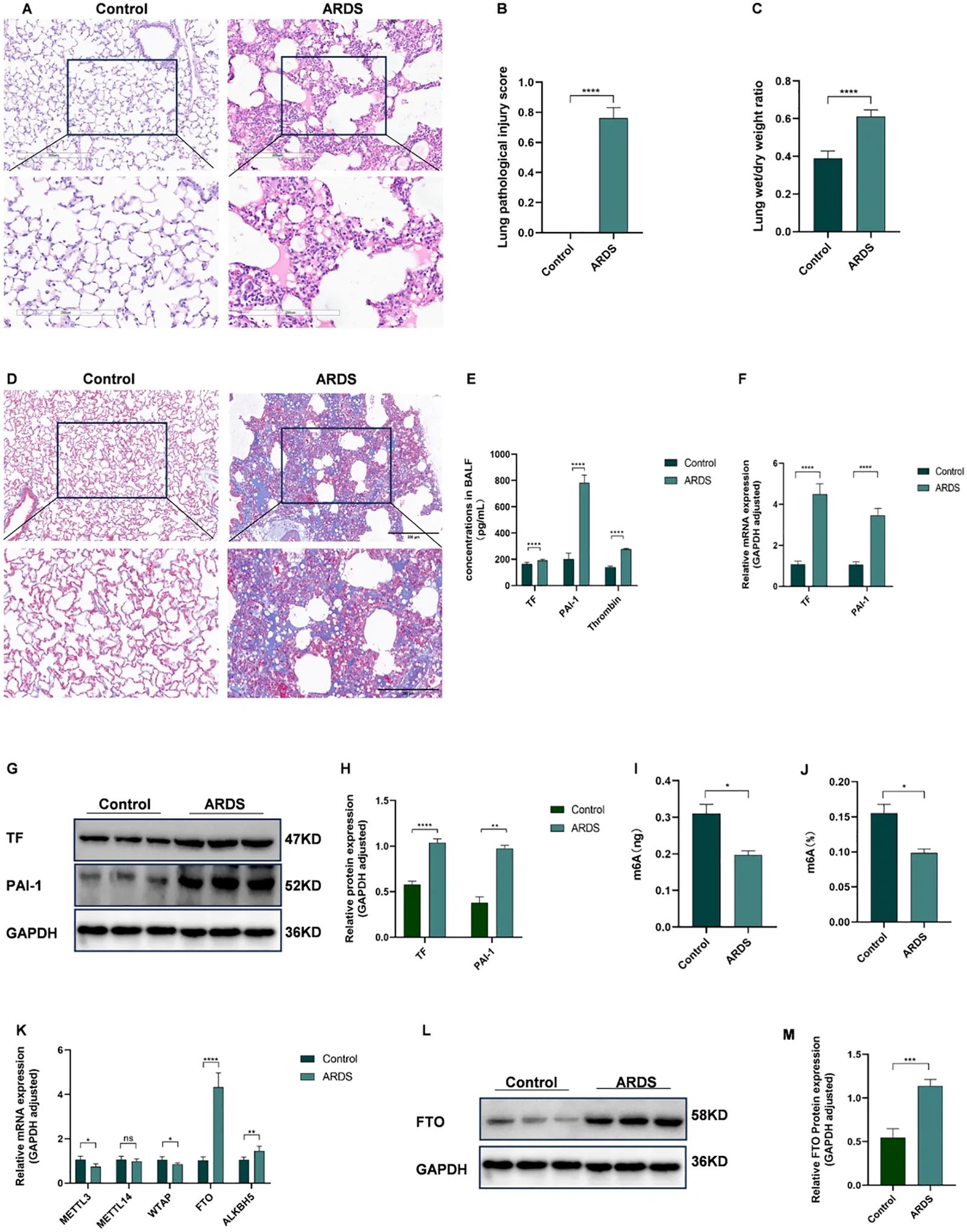
FTO is upregulated and associated with global m^6^A reduction in lipopolysaccharide-induced ARDS. **(A–C)** Representative histopathological images (hematoxylin and eosin staining) and quantitative analysis demonstrating diffuse alveolar damage and pulmonary edema induced by lipopolysaccharide (LPS).Scale bars: 200 &300µm. **(D)** Masson’s trichrome staining revealing collagen deposition (blue) in lung tissues. Scale bars: 200 &300µm. **(E)** Levels of TF, PAI-1, and thrombin in bronchoalveolar lavage fluid (BALF). **(F–H)** mRNA **(F)** and protein **(G, H)** expression levels of TF and PAI-1 in lung tissues. **(I–J)** Global m^6^A methylation levels in lung tissues quantified using a colorimetric assay. **(K–M)** mRNA **(K)** and protein **(L, M)** expression of the m^6^A demethylase FTO in lung tissues. Data are presented as mean ± SD. Statistical significance was determined by one-way ANOVA followed by Tukey’s *post hoc* test for multiple comparisons, or by two-tailed Student’s t-test for two-group comparisons. *p < 0.05, **p < 0.01, ***p < 0.001, ****p < 0.0001; ns, not significant (p > 0.05).

Consistent with these injury-related phenotypes, a marked reduction in global methylation was observed in lung tissues (**Figures 1I, J**). Screening of key regulatory genes revealed that the RNA demethylase FTO was the most significantly upregulated regulator at both transcriptional and translational levels (**Figures 1K–M**), implicating its potential involvement in mediating the observed hypomethylation.

### 3.2 FTO knockdown ameliorates lung injury and restores global methylation in LPS-induced ARDS

To investigate whether FTO directly contributes to the pathogenesis of ARDS, we performed lentivirus-mediated knockdown of FTO in rat lungs prior to LPS challenge (**Supplementary Figure S1**). FTO deficiency markedly attenuated LPS-induced lung injury, as evidenced by reduced pulmonary edema (**Figures 2A–C**). Concurrently, FTO knockdown potently suppressed the LPS-induced elevation of TF, PAI-1, and thrombin level in BALF (**Figure 2D**), downregulated collagen III expression in lung tissue (**Figures 2E, F**), and decreased both mRNA and protein levels of TF and PAI-1 (**Figures 2G–I**).

**Figure 2:**
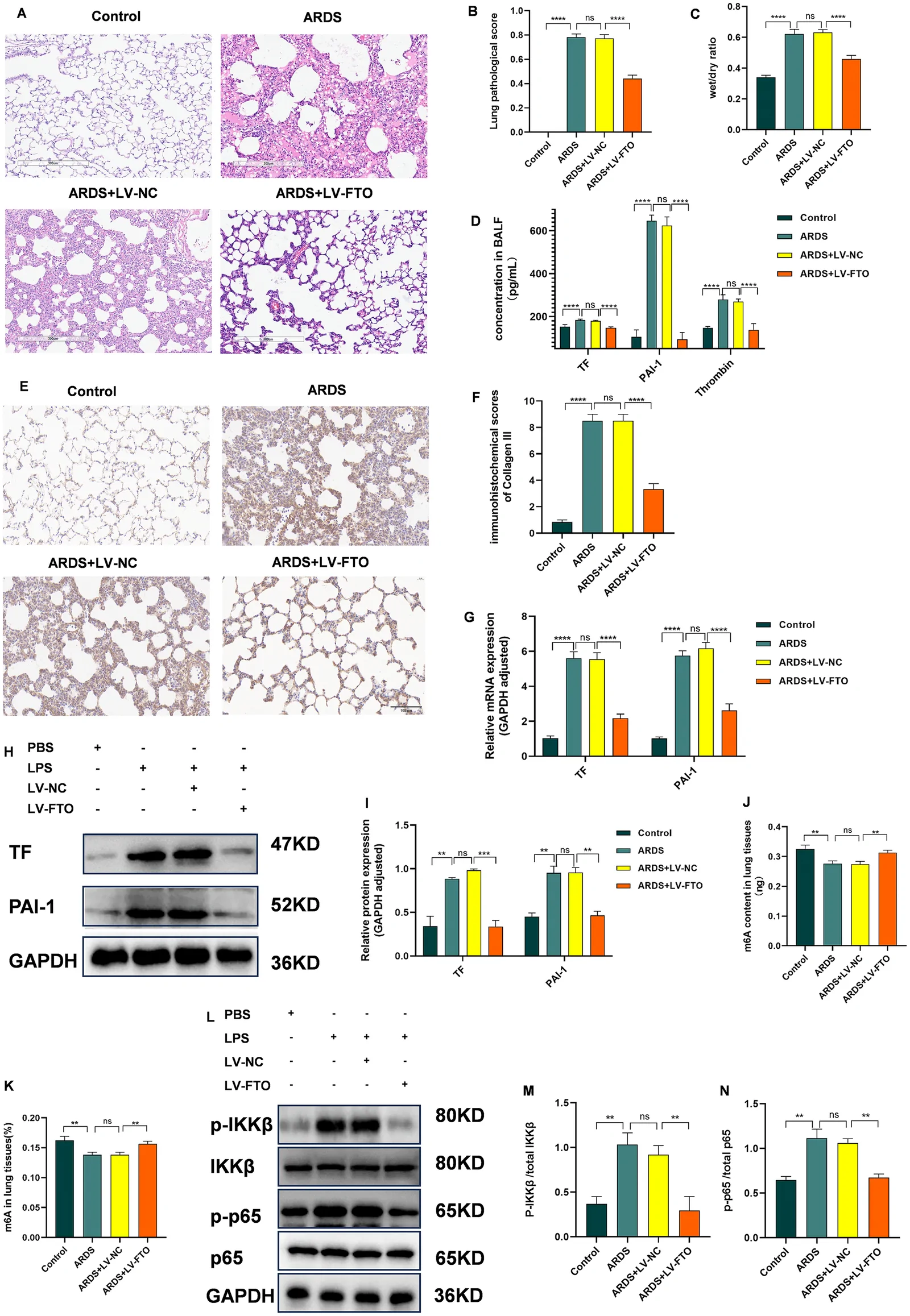
FTO knockdown attenuates lung injury, restores m^6^A methylation levels, and suppresses the NF-κB signaling pathway in lipopolysaccharide-induced ARDS. **(A–C)** Representative images and quantitative analysis of pulmonary edema, assessed by wet/dry weight ratio and histopathological evaluation, in rat lungs following FTO knockdown and LPS challenge. Scale bars: 200 µm. **(D)** Levels of TF, PAI-1, and thrombin in bronchoalveolar lavage fluid (BALF). **(E–F)** Expression of collagen III in lung tissue. Scale bars: 200 µm. **(G–I)** mRNA **(H)** and protein **(I, J)** expression of TF and PAI-1 in lung tissue. **(J, K)** Global m^6^A methylation levels in lung tissue. **(L–N)** Protein levels and phosphorylation status of key components of the NF-κB signaling pathway (IKKβ and p65) in lung tissue. Data are presented as mean ± SD. Statistical significance was determined by one-way ANOVA followed by Tukey’s *post hoc* test. (**p < 0.01, ***p < 0.001, ****p < 0.0001; ns, not significant (p > 0.05)). FTO knockdown was achieved via lentiviral delivery as detailed in **Supplementary Figure S1**.

At the epitranscriptomic level, FTO knockdown effectively restored global methylation levels that were diminished following LPS exposure (**Figures 2J, K**). Mechanistically, the protective effect of FTO silencing was associated with inhibition of the NF-κB signaling pathway, as indicated by reduced phosphorylation of IKKβ and p65 (**Figures 2L–N**). Collectively, these findings demonstrate that FTO acts as a key pathogenic driver in experimental ARDS.

### 3.3 Modulation of FTO directly regulates methylation and the gene expression of TF and PAI-1 in alveolar epithelial type II cells

To further validate the functional role of FTO at the cellular level, we performed lentivirus-mediated knockdown and overexpression of FTO in AEC II cells prior to LPS stimulation (**Supplementary Figure S2**). FTO knockdown effectively reversed the LPS-induced reduction in global methylation (**Figures 3A, B**) and concurrently suppressed the upregulation of TF and PAI-1 at both the mRNA (**Figure 3C**) and protein levels (**Figures 3D, E**). In contrast, FTO overexpression exacerbated the LPS-induced loss of global methylation (**Figures 3F, G**) and further enhanced the expression of TF and PAI-1 at mRNA (**Figure 3H**) and protein levels (**Figures 3I, J**).

**Figure 3:**
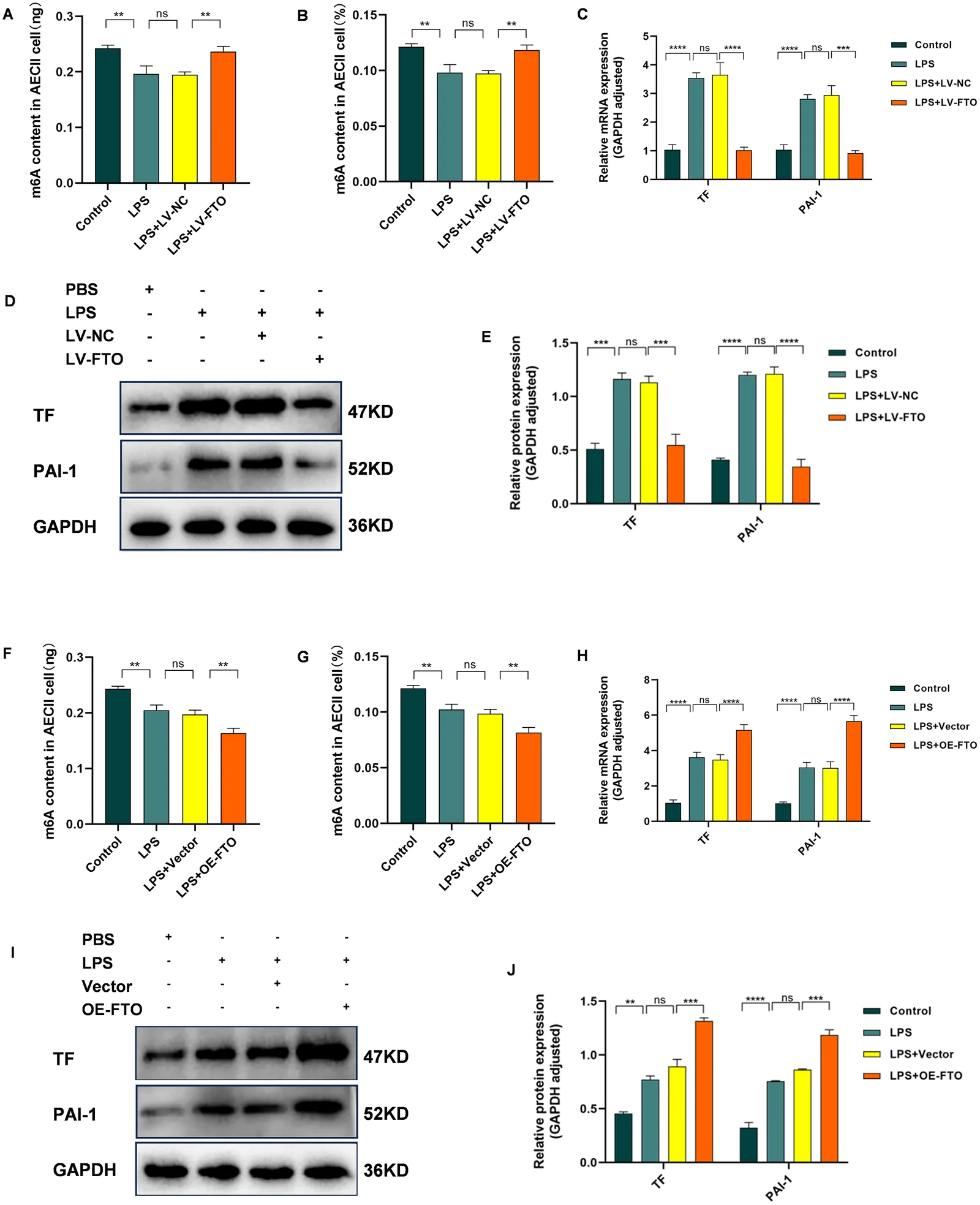
FTO regulates methylation and TF, PAI-1 gene expression in alveolar epithelial cells. **(A, B)** Global m^6^A methylation levels in alveolar epithelial type II (AEC II) cells following FTO knockdown under lipopolysaccharide (LPS) stimulation. **(C)** mRNA expression levels of TF and PAI-1 in FTO-knockdown (KD) cells with LPS treatment. **(D, E)** Representative western blot **(D)** and quantitative analysis **(E)** of TF and PAI-1 protein expression under the same conditions. **(F–G)** Global m^6^A methylation levels in AEC II cells following FTO overexpression (OE) under LPS stimulation. **(H)** mRNA expression levels of TF and PAI-1 in FTO-overexpressing (OE) cells with LPS treatment. **(I, J)** Representative western blot **(I)** and quantitative analysis **(J)** of TF and PAI-1 protein expression under the same conditions. Data are presented as mean ± SD. Statistical significance was determined by one-way ANOVA followed by Tukey’s *post hoc* test. (**p < 0.01, ***p < 0.001, ****p < 0.0001; ns, not significant (p > 0.05)). Lentivirus-mediated FTO knockdown and overexpression were performed as described in **Supplementary Figure S2**.

These complementary gain-and-loss-of-function experiments consistently demonstrate that FTO directly modulates methylation levels and promotes the expression of key procoagulant mediators in alveolar epithelial cells under inflammatory stress, thereby reinforcing its pathogenic role in LPS-induced ARDS.

### 3.4 PRKCB is identified as a key functional target of FTO

To identify downstream targets through which FTO exerts its procoagulant effects in ARDS, we performed integrative multi-omics analysis combining methylome (MeRIP-seq) and transcriptome (RNA-seq) profiling of lung tissues from control and LPS-treated rats. Differential analysis identified 3,181 differentially expressed genes (DEGs, |log_2_FC| ≥ 1, *p* < 0.05) and 927 differentially methylated genes (DMGs, |log_2_FC| ≥ 1, p < 0.05), with a significant overlap of 295 genes between the two datasets (**Figure 4A**). Saturation curve analysis confirmed that sequencing depth was sufficient for reliable peak detection; notably, the LPS group exhibited a leftward shift compared with controls, indicating a global reduction in methylation abundance in ARDS lung tissues (**Figure 4B**). Motif analysis revealed the canonical RRACH consensus sequence (where R = A/G, H = A/C/U) in both groups (**Figure 4C**), consistent with previously reported motifs (23).

**Figure 4:**
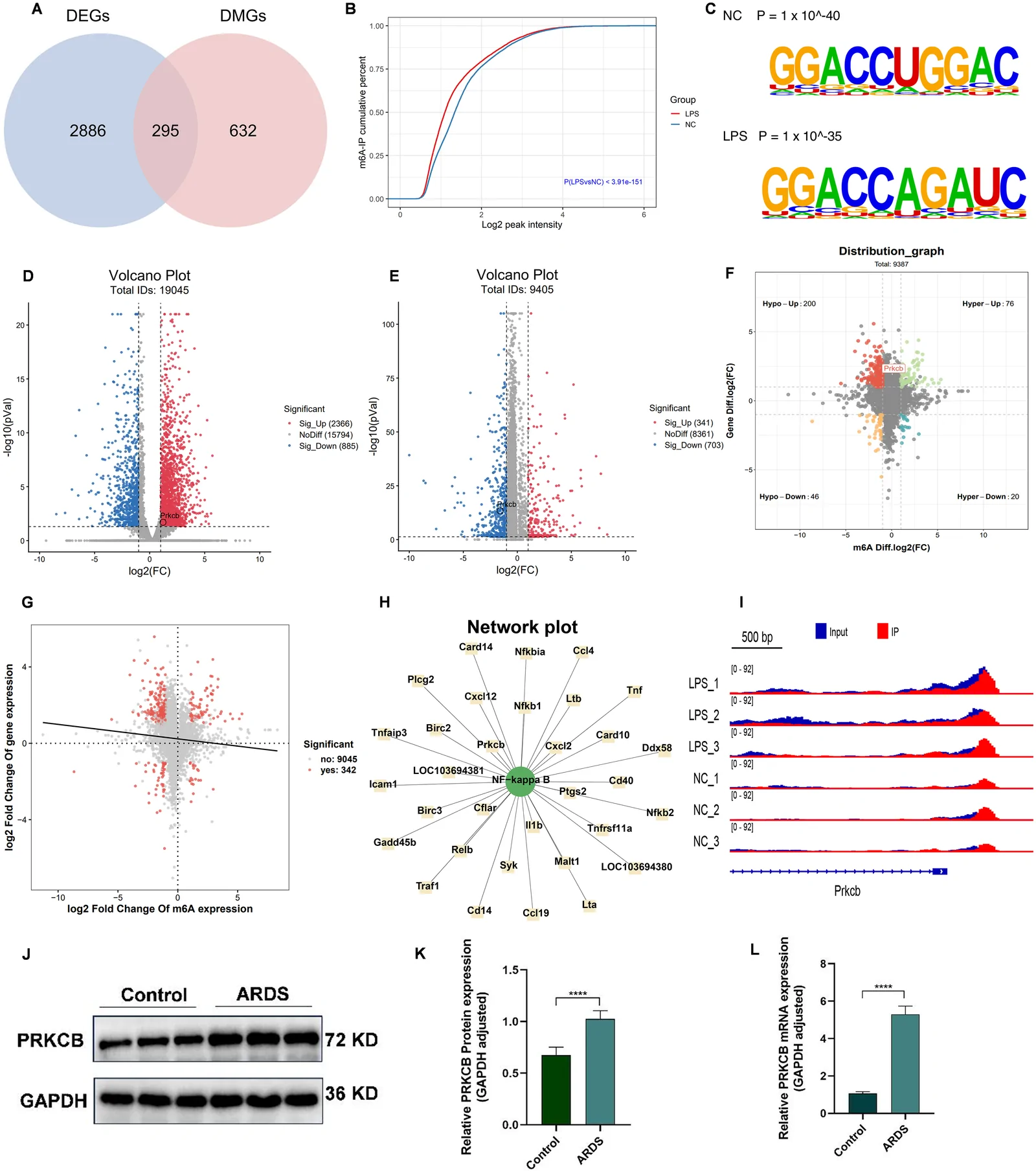
Integrative multi-omics analysis identifies PRKCB as a key m^6^A-hypomethylated gene in experimental ARDS. **(A)** Venn diagram showing the overlap between differentially m^6^A-methylated genes (DMGs) and differentially expressed genes (DEGs). **(B)** Saturation curve of m^6^A-seq reads, confirming sufficient sequencing depth for reliable peak detection. The leftward shift of the curve in the LPS group indicates a global reduction in m^6^A methylation abundance in ARDS lung tissues. **(C)** Consensus RRACH motif identified from m^6^A peaks in both control and ARDS groups. **(D)** Volcano plot of RNA-seq data showing genome-wide differential gene expression. PRKCB (red) is significantly upregulated at the transcriptional level in ARDS (∣log_2_FC∣≥1, *p* < 0.05). **(E)** Volcano plot of MeRIP-seq data showing differential m^6^A methylation peaks. The PRKCB peak (blue) exhibits significant m^6^A hypomethylation in ARDS, consistent with the global reduction shown in **(B)**. **(F)** Four-quadrant plot illustrating the relationship between m^6^A methylation changes (x-axis) and mRNA expression changes (y-axis). PRKCB (red) is located in the upper-left quadrant, characterized by hypomethylation and concomitant transcriptional upregulation. **(G)** Scatter plot showing a significant negative correlation between m^6^A methylation levels and mRNA expression across all m^6^A-modified transcripts, consistent with the role of m^6^A in promoting mRNA decay. **(H)** Network representation of the NF-κB signaling pathway highlighting PRKCB as a central regulatory node. **(I)** Integrative Genomics Viewer (IGV) tracks showing m^6^A peak profiles at the PRKCB locus in control and ARDS groups. **(J)** PRKCB mRNA expression in lung tissues determined by RT-qPCR. **(K, L)** Representative Western blots **(K)** and quantitative analysis **(L)** of PRKCB protein expression. Data are presented as mean ± SD. Statistical significance was determined by one-way ANOVA followed by Tukey’s *post hoc* test. ****p < 0.0001.

To identify genes whose loss correlated with transcriptional upregulation-a pattern characteristic of FTO-mediated demethylation-we cross-referenced the multi-omics datasets. Among the overlapping genes, we focused on those within the NF-κB signaling pathway, as our previous work established that NF-κB critically regulates alveolar procoagulant and fibrinolytic responses in ARDS (20). This selection was further supported by prior reports linking PRKCB to NF-κB activation in inflammatory lung injury (24). Volcano plots of RNA-seq (**Figure 4D**) and MeRIP-seq (**Figure 4E**) data highlighted PRKCB as a gene exhibiting both significant transcriptional upregulation and marked hypomethylation in ARDS. This relationship was further illustrated in the four-quadrant plot, where PRKCB resided in the upper-left quadrant characterized by decreased methylation and increased mRNA expression (**Figure 4F**). Correlation analysis across all -modified transcripts confirmed a significant negative association between methylation levels and mRNA expression (**Figure 4G**), consistent with the established role of in promoting mRNA decay.

Based on these multi-omics findings and the known role of PRKCB in inflammatory signaling, we prioritized PRKCB for further investigation. Pathway enrichment analysis positioned PRKCB as a central regulatory node within the NF-κB signaling network (**Figure 4H**). Integrative Genomics Viewer (IGV) visualization confirmed a pronounced reduction in peak density across the PRKCB transcript in ARDS samples (**Figure 4I**). Experimental validation by RT-qPCR and Western blot confirmed that both PRKCB mRNA (**Figure 4J**) and protein (**Figures 4K, L**) were significantly upregulated in ARDS lung tissues. Importantly, PRKCB has been previously reported to exacerbate lung injury through activation of the NF-κB pathway (24), suggesting its potential role as a critical molecular link connecting epitranscriptomic regulation to the pathogenesis of ARDS. Collectively, these data identify PRKCB as a key -hypomethylated and transcriptionally activated target of FTO in ARDS, warranting further mechanistic exploration.

### 3.5 FTO positively regulates PRKCB expression *in vivo* and *in vitro*

To further validate PRKCB as a critical functional downstream target of FTO, we conducted gain- and loss-of-function studies both *in vivo* and *in vitro*. In rats, lentivirus-mediated knockdown of FTO in the lungs prior to LPS challenge significantly suppressed the LPS-induced upregulation of PRKCB mRNA and protein levels in lung tissue (**Figures 5A–C**). In parallel, knockdown of FTO in AEC II cells *in vitro* before LPS stimulation similarly attenuated the LPS-induced elevation of PRKCB expression at both transcriptional and translational levels (**Figures 5D–F**). Conversely, overexpression of FTO in AEC II cells exacerbated the LPS-induced increase in PRKCB expression (**Figures 5G–I**).

**Figure 5:**
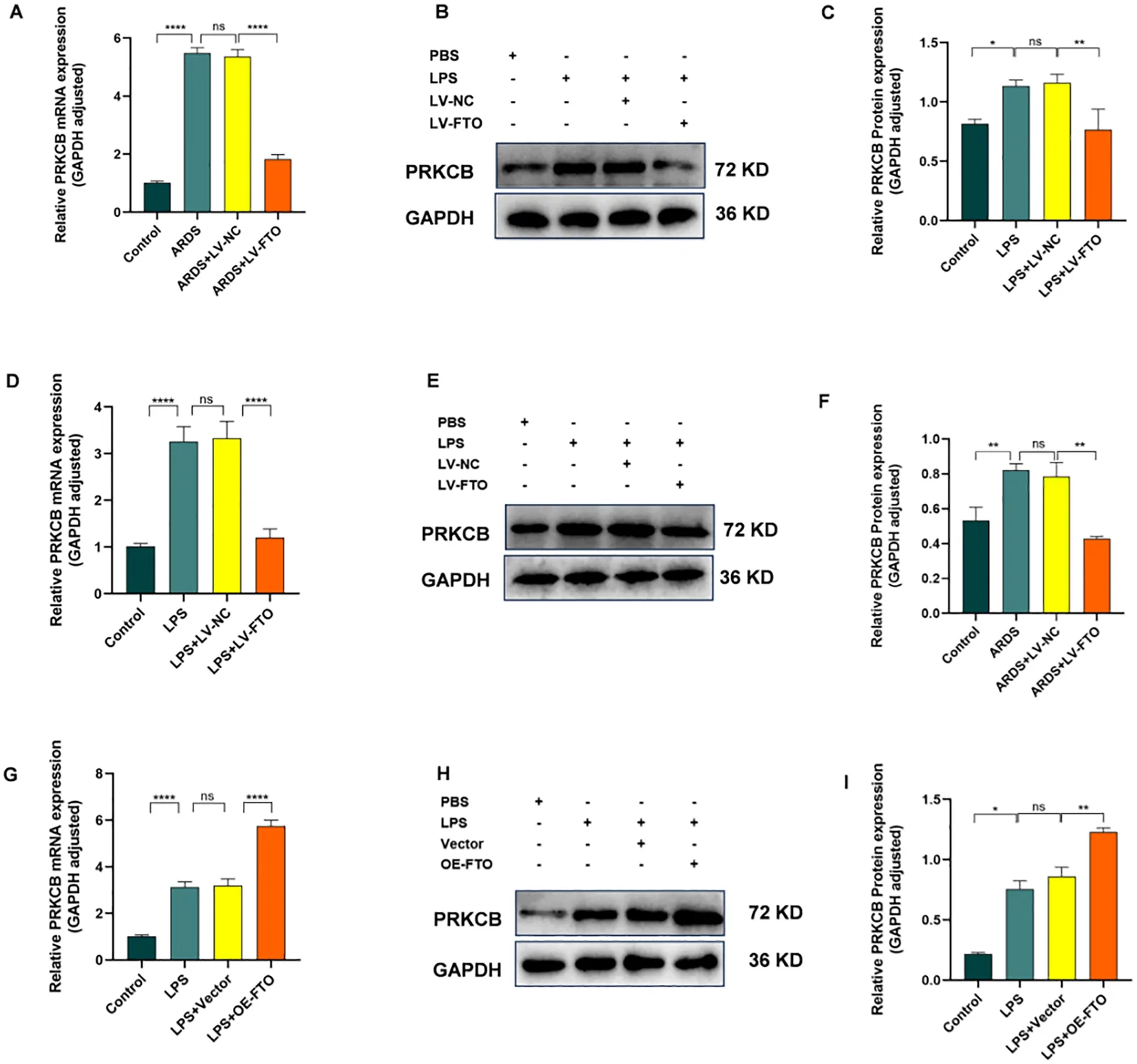
FTO positively regulates PRKCB expression *in vivo* and *in vitro*. **(A–C)** *In vivo* regulation by FTO knockdown. mRNA **(A)** and protein **(B, C)** levels of PRKCB in rat lung tissue following FTO knockdown and LPS challenge. **(D–F)** *In vitro* regulation by FTO knockdown in AEC II cells. mRNA **(D)** and protein **(E, F)** levels of PRKCB in cells subjected to FTO knockdown prior to LPS stimulation. **(G–I)** *In vitro* regulation by FTO overexpression in AEC II cells. mRNA **(G)** and protein **(H, I)** levels of PRKCB in cells subjected to FTO overexpression prior to LPS stimulation. Data are presented as mean ± SD. Statistical significance was determined by one-way ANOVA followed by Tukey’s *post hoc* test. *p < 0.05, **p < 0.01, ***p < 0.001, ****p < 0.0001; ns, not significant (p > 0.05).

### 3.6 FTO mediates the removal of modifications from PRKCB mRNA to enhance its stability

To elucidate the mechanistic link between FTO and PRKCB upregulation, we investigated whether FTO regulates PRKCB expression through -dependent mRNA stabilization. *In vivo*, methylated RNA immunoprecipitation followed by MeRIP-qPCR analysis revealed a significant reduction in methylation on PRKCB mRNA in LPS-injured lung tissues, which was reversed upon FTO knockdown (**Figure 6A**). Similarly, *in vitro* experiments showed that LPS stimulation decreased modification on PRKCB mRNA in AEC II cells, and this effect was also attenuated by FTO knockdown (**Figure 6B**). Conversely, FTO overexpression further reduced levels on PRKCB mRNA (**Figure 6C**).

**Figure 6:**
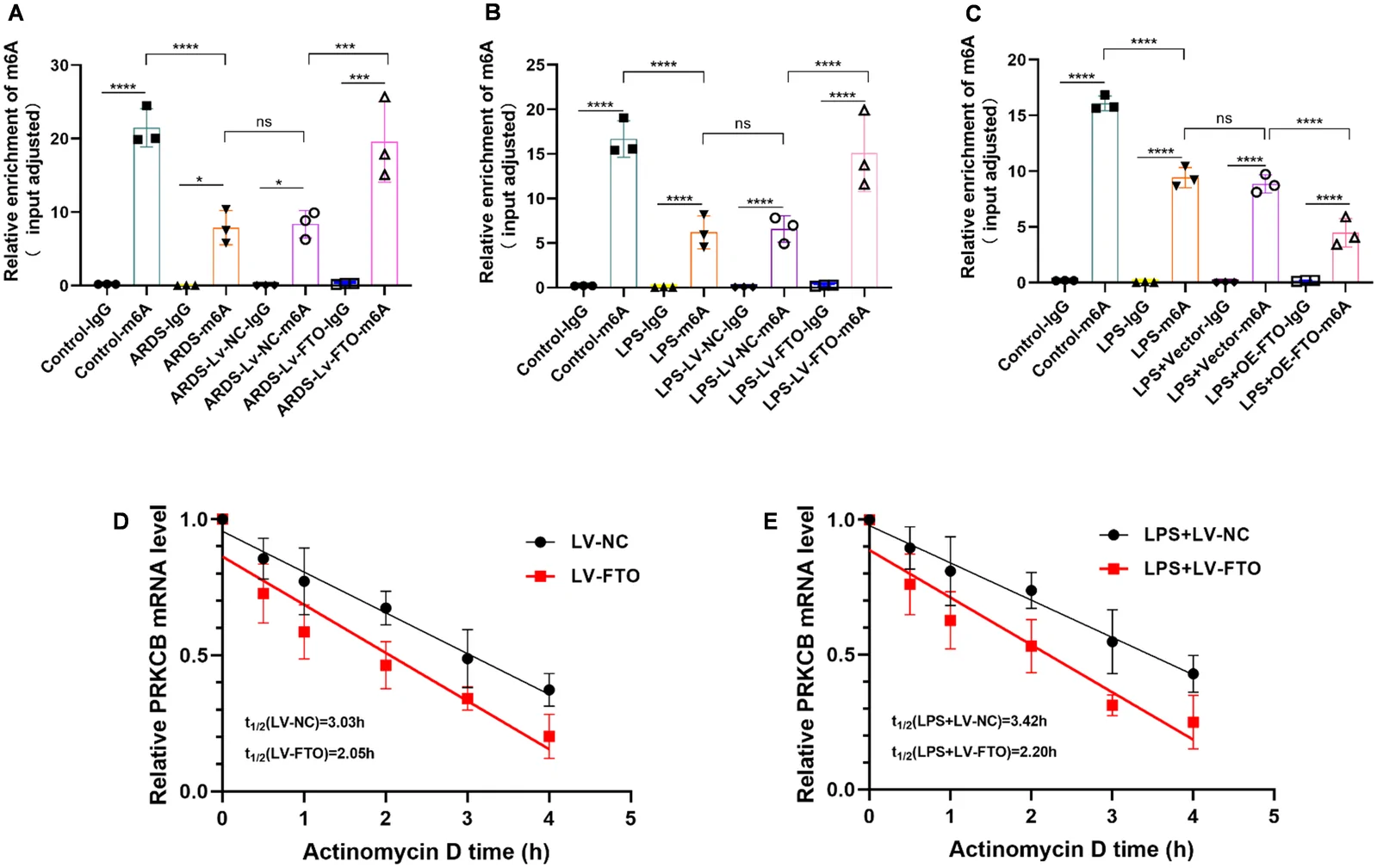
FTO removes m^6^A modifications from PRKCB mRNA, thereby enhancing its transcript stability. **(A)** enrichment levels on PRKCB mRNA in lung tissues from the indicated experimental groups, measured by MeRIP-qPCR. **(B)** levels on PRKCB mRNA in AEC II cells following FTO knockdown with or without LPS stimulation. **(C)** Effect of FTO overexpression on PRKCB methylation in AEC II cells under LPS challenge. **(D)** mRNA stability assay showing the half-life of PRKCB transcript in control versus FTO-knockdown AEC II cells after actinomycin D treatment. **(E)** Half-life of PRKCB mRNA in LPS-stimulated AEC II cells with or without FTO knockdown. Data are presented as mean ± SD. Statistical significance was determined by one-way ANOVA followed by Tukey’s *post hoc* test. *p < 0.05, ****p < 0.0001; ns, not significant (p > 0.05).

Given the well-established role of in regulating mRNA turnover, we performed actinomycin D chase assays to evaluate PRKCB transcript stability. FTO knockdown significantly shortened the half-life of PRKCB mRNA in AEC II cells under both basal and LPS-stimulated conditions (**Figures 6D, E**), indicating that FTO-mediated demethylation enhances PRKCB mRNA stability. This post-transcriptional regulatory mechanism provides a mechanistic basis for the observed upregulation of PRKCB protein expression.

### 3.7 PRKCB activates the NF-κB signaling pathway to promote the expression of TF and PAI-1

We next investigated the functional consequences of PRKCB upregulation (**Supplementary Figure S3**). In LPS-stimulated AEC II cells, PRKCB overexpression further enhanced the expression of TF and PAI-1 at both the mRNA (**Figure 7A**) and protein (**Figures 7B, C**) levels. In contrast, PRKCB knockdown markedly suppressed the mRNA (**Figure 7D**) and protein expression (**Figures 7E, F**) of these factors. Mechanistically, PRKCB was both necessary and sufficient for the full activation of the NF-κB signaling pathway. PRKCB overexpression robustly increased the phosphorylation of IKKβ and p65 (**Figures 7G–I**), whereas its knockdown markedly reduced the phosphorylation of these proteins (**Figures 7J–L**).

**Figure 7:**
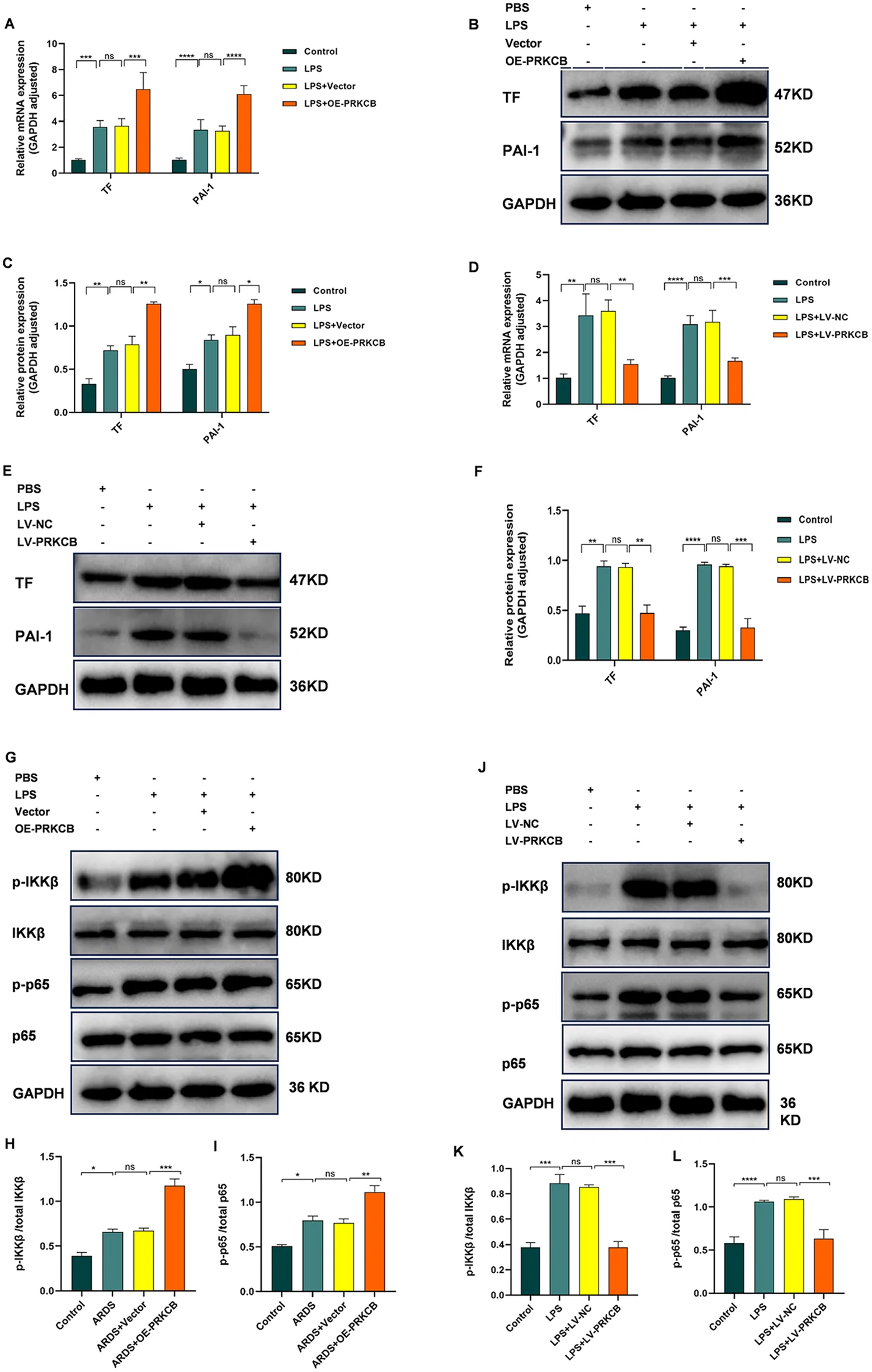
PRKCB is necessary and sufficient for driving TF and PAI-1 expression and NF-κB pathway activation. **(A–C)** Effect of PRKCB overexpression. mRNA **(A)** and protein **(B, C)** levels of TF and PAI-1 in LPS-stimulated AEC II cells following PRKCB overexpression. **(D–F)** Effect of PRKCB knockdown. mRNA **(D)** and protein **(E, F)** levels of TF and PAI-1 in LPS-stimulated AEC II cells following PRKCB knockdown. **(G–I)** PRKCB overexpression activates NF-κB. Representative western blots **(G)** and quantitative analysis of phosphorylated IKKβ **(H)** and p65 **(I)** in cells overexpressing PRKCB under LPS stimulation. **(J–L)** PRKCB knockdown inhibits NF-κB. Representative western blots **(J)** and quantitative analysis of phosphorylated IKKβ **(K)** and p65 **(L)** in cells with PRKCB knockdown under LPS stimulation. Data are presented as mean ± SD. Statistical significance was determined by one-way ANOVA followed by Tukey’s *post hoc* test for multiple comparisons, or by two-tailed Student’s t-test for two-group comparisons. *p < 0.05, **p < 0.01, ***p < 0.001, ****p < 0.0001; ns, not significant (p > 0.05).

### 3.8 Pharmacological validation of PRKCB as an upstream regulator of NF-κB activation

To rigorously evaluate whether PRKCB regulates TF and PAI-1 through the NF-κB signaling pathway, we utilized pharmacological interventions. Treatment with benzoxathiole, a specific NF-κB inhibitor, abolished the upregulation of both TF and PAI-1 at the mRNA and protein levels induced by PRKCB overexpression, without altering PRKCB cellular immunofluorescence analysis demonstrated cytoplasmic co-localization of PRKCB and phosphorylated IKKβ (p-IKKβ) expression itself (**Figures 8A–C**). Conversely, administration of HY-134476, an NF-κB agonist, reversed the downregulation of TF and PAI-1 caused by PRKCB knockdown (**Figures 8D–F**).

**Figure 8:**
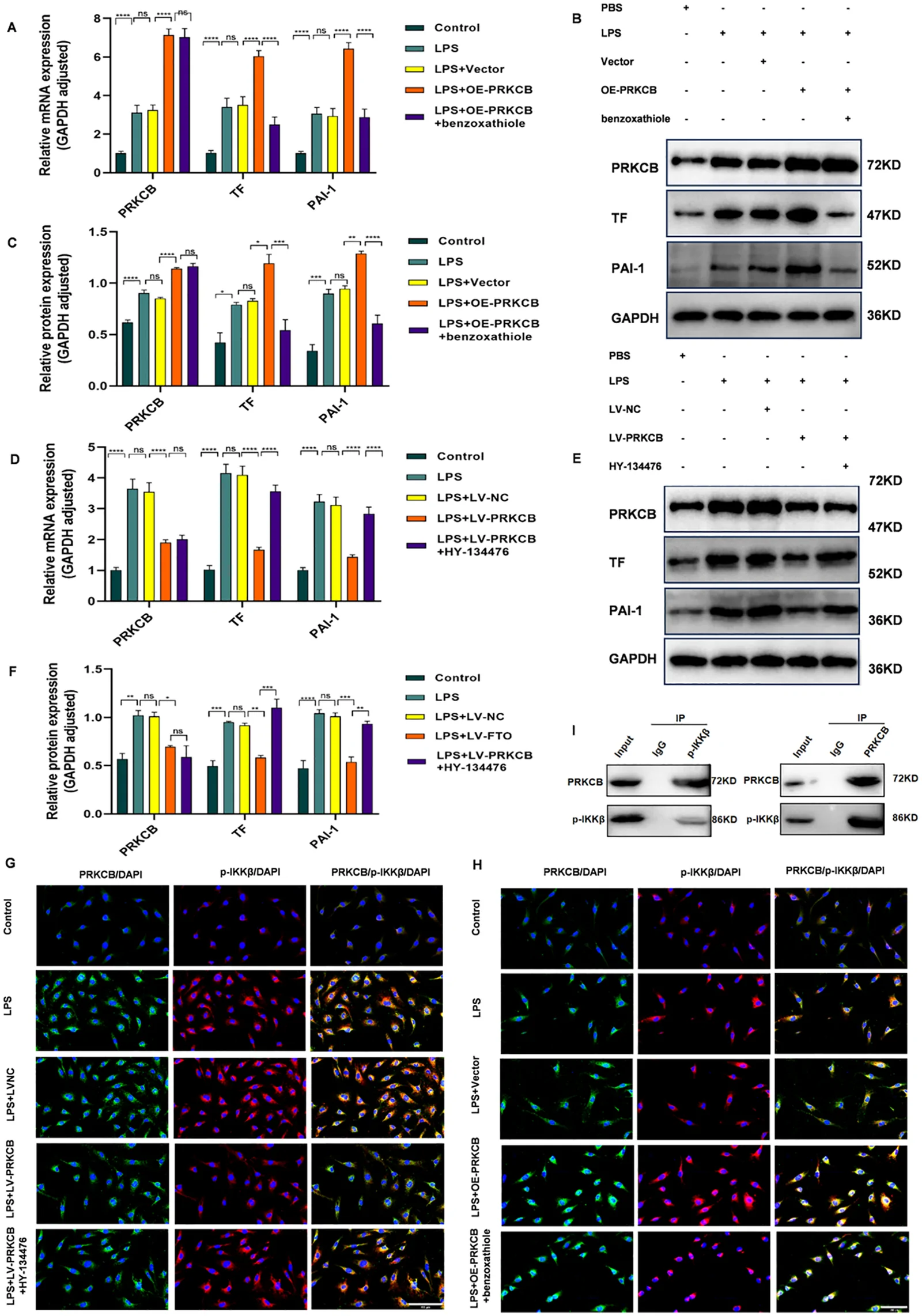
PRKCB acts upstream of NF-κB to regulate procoagulant factor expression. **(A–C)** NF-κB inhibition reverses PRKCB overexpression phenotypes. **(A)** mRNA levels of TF and PAI-1, **(B)** representative western blots, and **(C)** quantitative analysis of protein expression in LPS-stimulated AEC II cells under indicated conditions (PRKCB overexpression ± benzoxathiole). **(D–F)** NF-κB activation rescues PRKCB knockdown phenotypes. **(D)** mRNA levels, **(E)** representative western blots, and **(F)** quantitative protein analysis in LPS-stimulated AEC II cells under indicated conditions (PRKCB knockdown ± HY-134476). **(G, H)** Analysis of PRKCB and p-IKKβ co-localization by immunofluorescence. **(G)** Representative images showing co-localization (yellow) of PRKCB (green) and p-IKKβ (red) under PRKCB knockdown conditions, with or without HY-134476 treatment. **(H)** Representative images showing co-localization under PRKCB overexpression conditions, with or without benzoxathiole treatment. Nuclei are stained with DAPI (blue). Scale bars: 100 µm. **(I)** Co-immunoprecipitation (co-IP) assay demonstrating interaction between PRKCB and p-IKKβ. Lysates from LPS-stimulated AEC II cells were immunoprecipitated with an anti-PRKCB antibody or control IgG, followed by immunoblotting for p-IKKβ and PRKCB. Data are presented as mean ± SD. Statistical significance was determined by one-way ANOVA followed by Tukey’s *post hoc* test for multiple comparisons, or by two-tailed Student’s t-test for two-group comparisons.*p < 0.05, **p < 0.01, ***p < 0.001, ****p < 0.0001; ns, not significant (p > 0.05).

In LPS-stimulated AEC II cells, this co-localization was diminished upon PRKCB knockdown and restored by HY-134476 treatment (**Figure 8G**). In contrast, benzoxathiole suppressed the enhanced co-localization observed with PRKCB overexpression (**Figure 8H**). Moreover, co-immunoprecipitation (co-IP) assays confirmed a direct physical interaction between PRKCB and p-IKKβ in LPS-stimulated AEC II cells (**Figure 8I**). Taken together, these findings consistently position PRKCB as an upstream regulator of NF-κB activation.

### 3.9 Genetic rescue studies validate the FTO–PRKCB–NF-κB signaling axis

Finally, we conducted genetic rescue experiments to validate the linear signaling pathway linking FTO to alveolar hypercoagulability and impaired fibrinolysis. In cells overexpressing FTO, concurrent knockdown of PRKCB effectively reversed the upregulation of TF and PAI-1 at both mRNA (**Figure 9A**) and protein levels (**Figures 9B, C**), and attenuated NF-κB hyperactivation (**Figures 9G–I**). Conversely, in FTO-knockdown cells, simultaneous overexpression of PRKCB restored TF and PAI-1 expression at both mRNA and protein levels to those observed under LPS-induced injury conditions (**Figures 9D–F**), and concomitantly rescued NF-κB activity (**Figures 9J–L**). Collectively, these findings firmly establish PRKCB as the key downstream mediator through which FTO exerts its procoagulant and antifibrinolytic effects in ARDS.

**Figure 9:**
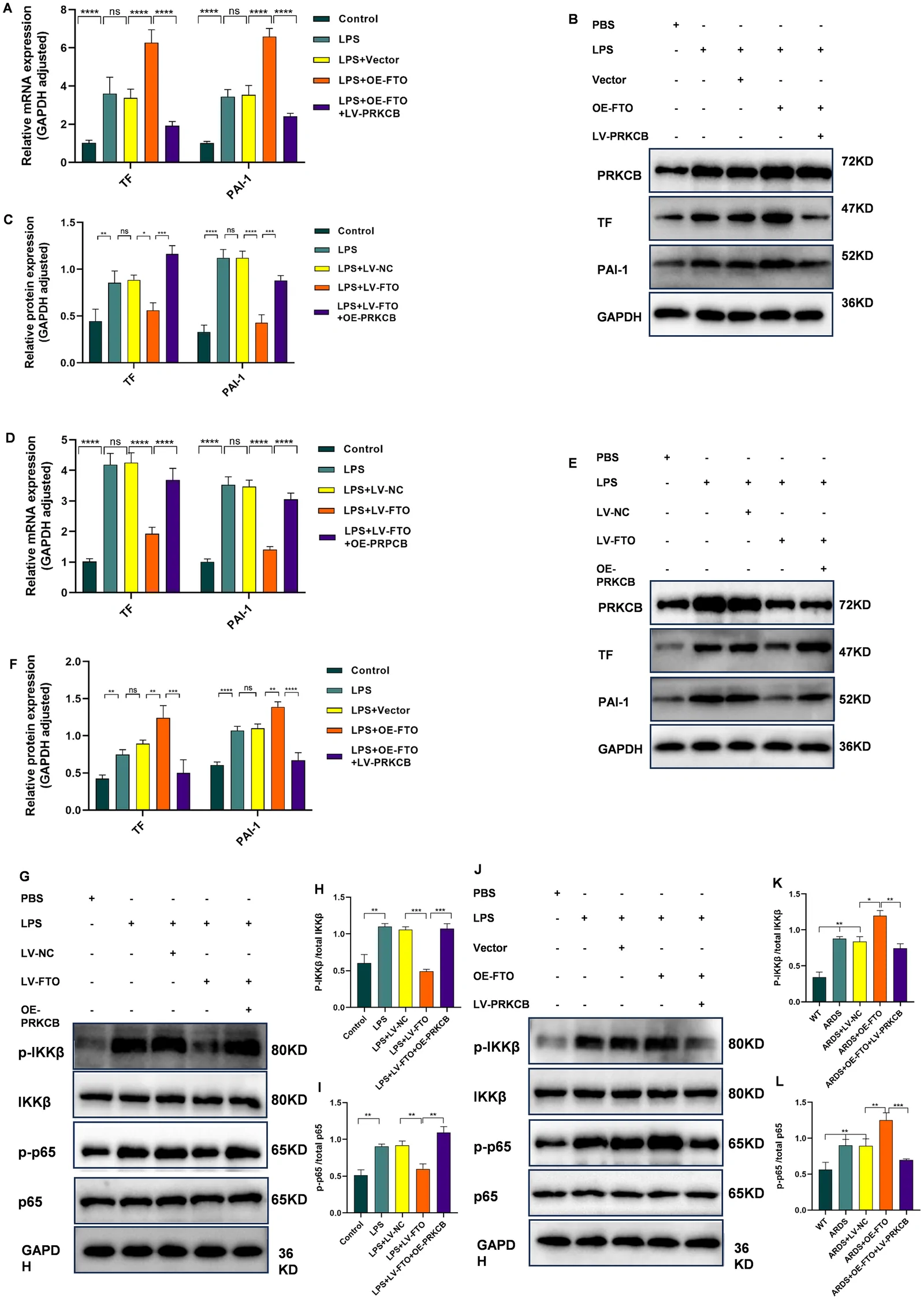
PRKCB mediates the procoagulant effects of FTO in alveolar epithelial cells. **(A–C)** PRKCB knockdown rescues FTO overexpression phenotypes. **(A)** mRNA levels of TF and PAI-1, **(B)** representative western blots, and **(C)** quantitative protein analysis in LPS-stimulated AEC II cells under indicated conditions (FTO overexpression ± PRKCB knockdown). **(D–F)** PRKCB overexpression reverses FTO knockdown phenotypes. **(D)** mRNA levels, **(E)** representative western blots, and **(F)** quantitative protein analysis in LPS-stimulated AEC II cells under indicated conditions (FTO knockdown ± PRKCB overexpression). **(G–I)** NF-κB pathway activity under conditions in A-C. **(G)** Representative western blots and quantitative analysis of phosphorylated IKKβ **(H)** and p65 **(I)**. **(J–L)** NF-κB pathway activity under conditions in D-F. **(J)** Representative western blots and quantitative analysis of phosphorylated IKKβ **(K)** and p65 **(L)**. Data are presented as mean ± SD. Statistical significance was determined by one-way ANOVA followed by Tukey’s *post hoc* test for multiple comparisons, or by two-tailed Student’s t-test for two-group comparisons.*p < 0.05, **p < 0.01, ***p < 0.001, ****p < 0.0001; ns, not significant (p > 0.05).

## 4 Discussion

Our study elucidates a previously unrecognized epitranscriptomic pathway that critically regulates the dysregulated coagulation and fibrinolytic balance in ARDS. We demonstrate that the m^6^A demethylase FTO is upregulated in injured alveolar epithelial cells, where it promotes pathogenesis by removing m^6^A modifications from PRKCB mRNA, thereby enhancing its transcript stability and protein expression. The resulting increase in PRKCB protein levels activates the NF-κB signaling pathway, ultimately driving the expression of TF and PAI-1, the two most important factors fostering a pro-fibrinolytic alveolar microenvironment (**Figures 6**, **7**). This FTO-PRKCB-NF-κB axis constitutes a mechanistic link between epitranscriptomic regulation and the hallmark pathological feature of alveolar fibrin deposition in ARDS.

The discovery of significantly decreased global m^6^A levels in the lungs of ARDS rats highlights a profound dysregulation of the epitranscriptome in this condition. The specific and pronounced upregulation of the m^6^A “eraser” FTO positions it as a likely master regulator of this hypomethylated landscape. Although FTO has been implicated in other inflammatory conditions, such as septic shock and macrophage activation (25, 26), its role in ARDS-particularly in regulating the alveolar coagulation cascade-has remained unexplored. Our functional data, derived from both *in vivo* knockdown and *in vitro* overexpression models, unequivocally establish FTO as a pathogenic driver that is essential for the full manifestation of the injury phenotype. This expands the known functional repertoire of FTO beyond cancer and metabolic disorders (27), underscoring its critical role in critical illness.

Beyond ARDS, FTO has been implicated in other pulmonary diseases. In lung cancer, FTO promotes tumor progression by regulating oncogene expression through -dependent mechanisms (28). In pulmonary fibrosis, modifications participate in fibroblast activation and epithelial-mesenchymal transition, though the role of FTO appears complex and cell-type-dependent (29).These observations suggest that FTO exerts both disease-specific and context-dependent effects in the lung-functioning as a pathogenic driver in ARDS and cancer, while potentially playing a complex role in fibrotic conditions-underscoring the importance of cell-type and disease-stage-specific considerations when targeting FTO therapeutically.

The identification of PRKCB as a key functional target of FTO through our integrated MeRIP-seq and RNA-seq approach represents a pivotal finding. Although previous studies have suggested a role for PKC signaling in lung injury, primarily based on experiments using broad-spectrum inhibitors (30), the precise molecular effector and its upstream regulatory mechanism have remained undefined. Our study establishes a clear mechanistic cascade: FTO directly binds to and demethylates PRKCB mRNA, leading to reduced m^6^A modification, which enhances transcript stability by limiting recognition by RNA decay factors such as YTHDF2; this, in turn, increases PRKCB protein expression and drives NF-κB activation. This FTO-PRKCB-NF-κB axis provides a compelling example of how a single epitranscriptomic modification can amplify pro-inflammatory and pro-coagulant signaling cascades at the post-transcriptional level. The exact molecular mechanism by which PRKCB activates IKKβ remains to be fully elucidated. We hypothesize that PRKCB may directly phosphorylate IKKβ at specific serine residues or act via intermediate kinases, thereby lowering the threshold for NF-κB activation in response to LPS stimulation. This constitutes a promising direction for future investigation.

While our study focused on NF-κB, FTO has been reported to regulate other signaling pathways including PI3K/AKT, MAPK, and JAK/STAT in various disease models (31). Whether FTO engages these pathways in alveolar epithelial cells during ARDS, and whether such engagement contributes to the procoagulant phenotype beyond NF-κB activation, warrants further investigation.

Our findings should be interpreted within the broader and rapidly evolving context of epitranscriptomics in lung pathophysiology. For instance, Reem Faraj et al. identified METTL3 (methyltransferase-like 3), a selective RNA methyltransferase for, as a central regulator of proinflammatory gene expression in acute lung injury (ALI), and proposed that targeting METTL3 could offer a promising drug discovery strategy for ALI treatment (16). Further supporting this, multiple studies have elucidated specific mechanisms through which methylation influences ALI: METTL3 inhibition was shown to alleviate LPS-induced alveolar epithelial cell apoptosis and lung injury by restoring neprilysin expression (32); modulation of -modified transcription factor GATA6 was found to affect epithelial cytokines in ALI (17); neutrophil extracellular traps were reported to impair autophagic flux via METTL3 in sepsis-associated ALI (33); METTL4-mediated was shown to promote lung injury by activating ferroptosis in alveolar epithelial cells (34). Additionally, LncRNA THRIL, transcriptionally activated by AP-1 and stabilized by METTL14-dependent modification, exacerbates LPS-evoked alveolar epithelial injury (35). Moreover, modifications mediated by METTL3 can aggravate ferroptosis through -IGF2BP2-dependent mitochondrial metabolic reprogramming in sepsis-induced ALI (11), and neutrophil extracellular traps have also been linked to -associated ferroptosis activation in alveolar epithelial cells during sepsis-related lung injury (13).

While METTL3 was downregulated in ARDS lung tissues, it is the balance between writers and erasers-not any single regulator-that determines net levels. In our model, FTO upregulation overwhelmingly drives demethylation, overriding the modest METTL3 reduction. Furthermore, PRKCB mRNA stability is not solely determined by METTL3 activity; the FTO-mediated removal of marks, particularly at specific sites that recruit decay-promoting readers such as YTHDF2, is the primary determinant of PRKCB transcript turnover.

Our study builds upon and extends these earlier reports in three key respects. First, we identified FTO-rather than ALKBH5 or METTL3-as the predominant epitranscriptomic regulator in alveolar epithelial type II (AEC II) cells that controls the coagulation-fibrinolysis balance, underscoring the cell-type-specific nature of the RNA modification machinery. Second, we delineate alveolar coagulopathy as a core pathophysiological process that is fundamentally regulated by m^6^A methylation, thereby expanding the known functional scope of epitranscriptomic control beyond classical inflammatory cytokine signaling. Third, we map a complete signaling cascade from an RNA demethylase (FTO) to a specific kinase (PRKCB) and ultimately to a master transcriptional regulator (NF-κB), offering a comprehensive mechanistic framework that is often lacking in studies focused on RNA modifications.

The therapeutic implications of our findings are significant. Current management of ARDS remains largely supportive, consisting of lung-protective mechanical ventilation, prone positioning, and extracorporeal membrane oxygenation (ECMO) in severe cases. Despite these interventions, mortality remains unacceptably high, with reported rates exceeding 40% in severe ARDS (36). Pharmacological therapies targeting specific pathogenic pathways have repeatedly failed in clinical trials, largely due to the heterogeneous nature of ARDS and the complex interplay between inflammation and coagulation. The recognition that alveolar fibrin deposition-driven by the convergence of hypercoagulation and impaired fibrinolysis-is a core pathological feature of ARDS highlights the need for therapeutic strategies that target the inflammation-coagulation crosstalk. Our identification of the FTO-PRKCB-NF-κB axis as a unifying epitranscriptomic regulator of both inflammatory and procoagulant responses suggests that targeting this axis may offer advantages over conventional approaches that address only one arm of the pathophysiological cascade.

Given the repeated failure of targeted therapies in ARDS, the FTO-PRKCB axis represents a novel and promising therapeutic target. The development of potent and selective FTO inhibitors is already an active area of investigation in oncology, and our data provide a strong rationale for evaluating these agents in preclinical models of ARDS. Likewise, PRKCB-specific inhibitors, which have been explored in other disease contexts such as diabetic complications, may be strategically repurposed for lung injury. While intratracheal aerosolized lentivirus delivery is feasible for proof-of-concept studies, clinical translation faces challenges including immunogenicity, insertional mutagenesis risk, and aerosol biosafety concerns. Small-molecule FTO inhibitors-including bisantrene (currently in Phase II trials for AML, NCT04989335), FB23-2, and Dac51-currently under development for oncology may offer a more clinically tractable strategy, particularly via aerosolized administration for local pulmonary delivery (37). Targeting this axis may offer advantages over conventional anticoagulant strategies by simultaneously attenuating both the pro-coagulant state and the underlying inflammatory response, potentially disrupting the self-amplifying cycle between coagulation and inflammation in the alveolar compartment.

Our study has several limitations. The upstream signals responsible for FTO upregulation in ARDS remain to be fully elucidated; potential candidates include NF-κB itself, which may form a feed-forward regulatory loop, or hypoxia-inducible factors (HIFs). Furthermore, although our investigation focused on PRKCB, it is likely that FTO regulates a broader network of mRNAs that collectively contribute to the ARDS phenotype. Future studies utilizing FTO cross-linking immunoprecipitation sequencing (FTO-CLIP) or related approaches will be essential to comprehensively map its RNA target landscape in this pathological context. Additionally, the downstream reader proteins that recognize the demethylated sites on PRKCB mRNA remain to be definitively identified. While our data demonstrate that FTO-mediated demethylation enhances PRKCB mRNA stability (**Figures 6D, E**), the precise reader protein involved in this process has not been formally examined. Future studies employing RNA immunoprecipitation (RIP)-qPCR to detect YTHDF2 binding to PRKCB mRNA, as well as YTHDF2 knockdown or overexpression experiments, are warranted to fully elucidate this epitranscriptomic regulatory cascade. Such investigations may also reveal whether other readers, such as YTHDF1 (which promotes translation) or IGF2BPs (which stabilize mRNA), contribute to the FTO-PRKCB axis in a context-dependent manner. Finally, the translational relevance of our findings requires validation in human clinical samples and more physiologically complex disease models.

In conclusion, we have delineated a linear FTO/m^6^A-PRKCB-NF-κB signaling axis that serves as a critical driver of alveolar fibrin deposition in acute respiratory distress syndrome (ARDS). This study underscores the epitranscriptome as a pivotal regulatory layer in critical illness and identifies FTO as a promising therapeutic target for mitigating the severe coagulopathy that characterizes this condition.

## Data Availability

The data have been deposited in GEO under accession number GSE316047 and are publicly available. The link is: https://www.ncbi.nlm.nih.gov/geo/query/acc.cgi?acc=GSE316047.
