# Supplementary material for: FTO-mediated demethylation of PRKCB mRNA activates NF-κB to trigger alveolar fibrin deposition in ARDS
Source: Front Immunol. 2026 Oct 14;17:1960313. doi: 10.3389/fimmu.2026.1960313 (PMC13684130; doi:10.3389/fimmu.2026.1960313)
Supplement: Supplementary file 1 [file DataSheet1.pdf]

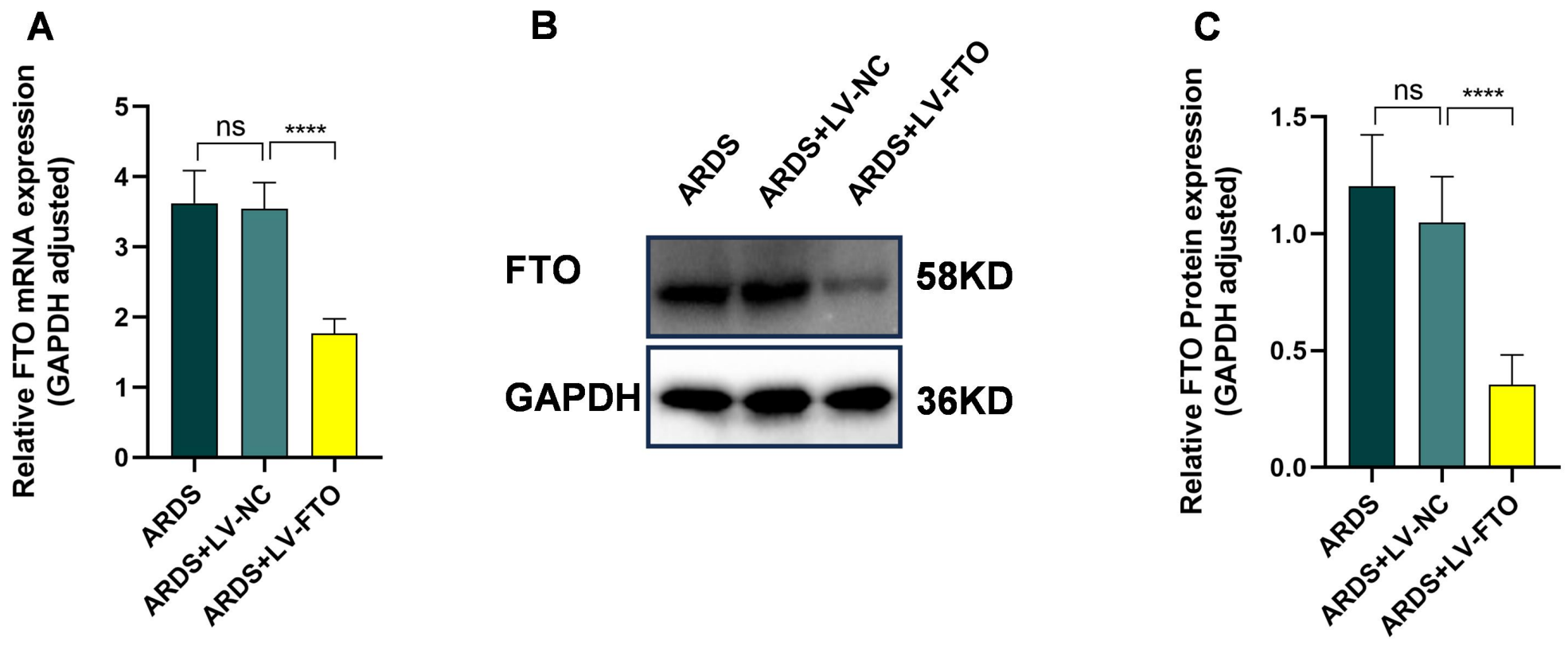

Figure S1. FTO expression in lung tissues from experimental rat groups. (A), FTO mRNA levels across groups, measured by qRT-PCR. (B), Representative western blot images of FTO protein expression in lung tissue lysates and Semiquantitative analysis of FTO protein levels (C). Data are presented as mean±SD. Statistical significance was determined by one-way ANOVA followed by Tukey's post hoc test for multiple comparisons, or by two-tailed Student's t-test for two-group comparisons. ns, not significant ($p > 0.05$); ****$p < 0.0001$.

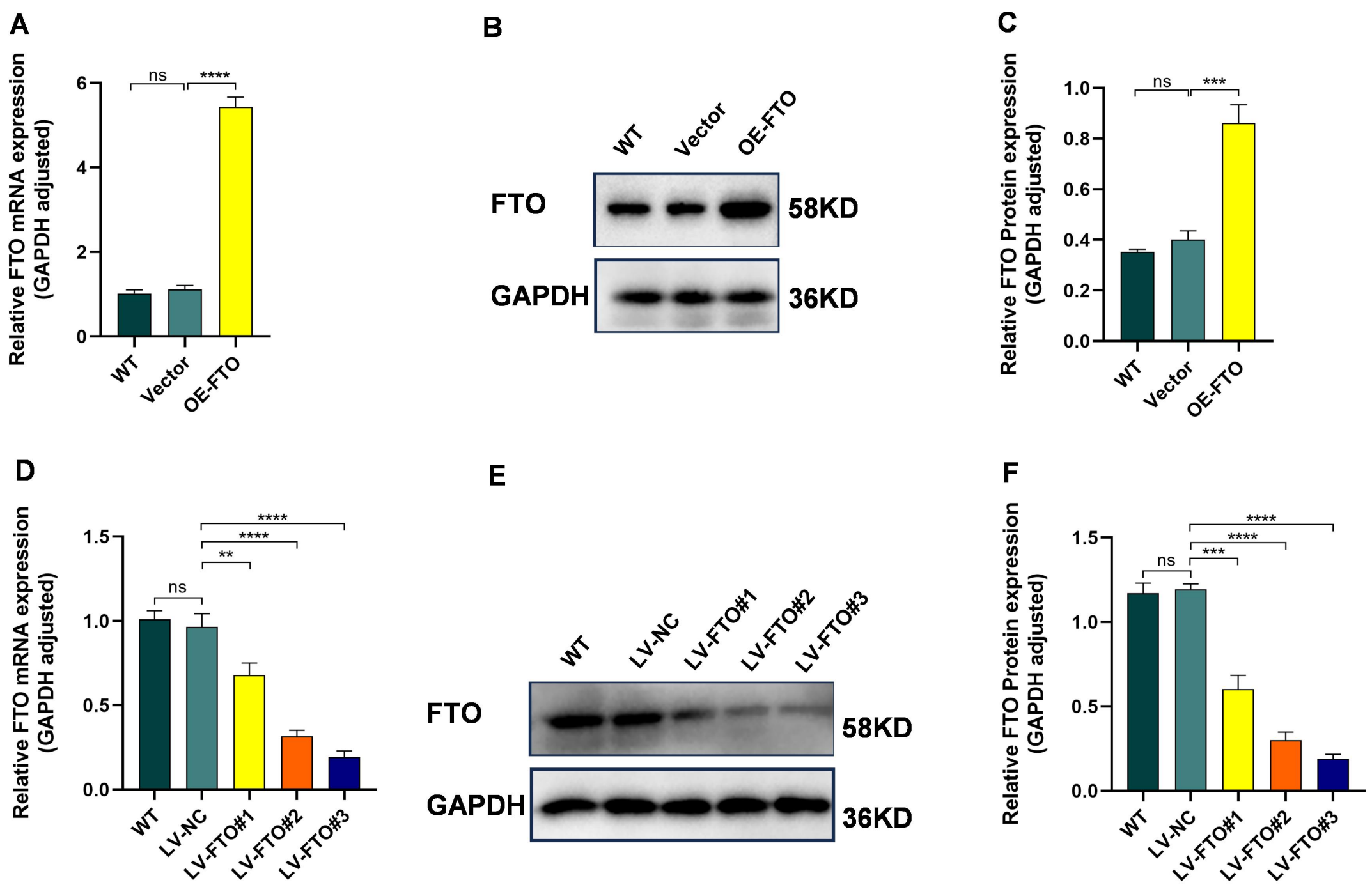

Figure S2. Validation of FTO overexpression and knockdown efficiencies in RLE-6TN cells. (A-C), FTO levels in the overexpression model. (A)**,** mRNA levels measured by qRT-PCR. (B), Representative western blot images of FTO protein expression in lung tissue and Semiquantitative analysis of FTO protein levels (C). (D-F), FTO levels in the knockdown model. (D), mRNA levels measured by qRT-

PCR. (E), Representative western blot of FTO protein. (F), Quantification of FTO protein expression. Data are presented as mean ± s.d. (n=3 independent experiments). Statistical significance was determined by one-way ANOVA with Tukey's post hoc test. ns, not significant (*p*>0.05); **p*<0.05; ***p*<0.01; ****p*<0.001; *****P*<0.0001. Abbreviations: WT, wild-type; Vector, empty vector control; OE-FTO, FTO-overexpressing; LV-NC, non-targeting shRNA control; LV-FTO#1/2/3, FTO-targeting shRNA (sites #1, #2, #3).

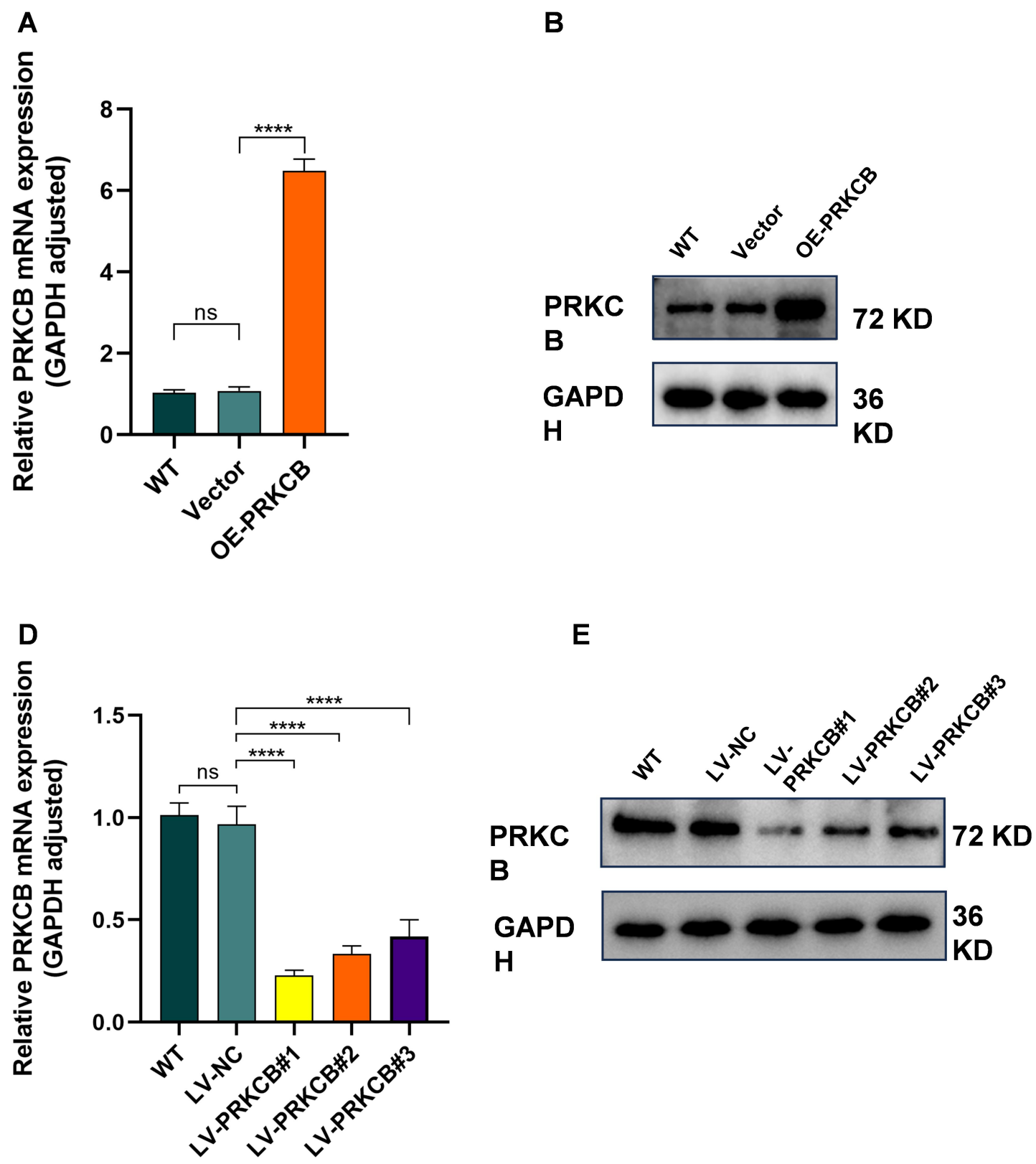

Figure S3. Validation of PRKCB overexpression and knockdown efficiencies in RLE-6TN cells. (A-C), PRKCB levels following overexpression. (A), mRNA levels by qRT-PCR. (B), Representative western blot. (C), Quantification of protein expression. (D-F), PRKCB levels following knockdown. (D), mRNA levels by qRT-PCR. (E), Representative western blot. (F), Quantification of protein expression. Data are presented as mean ± s.d. (n=3 independent experiments). Statistical significance was determined by one-way ANOVA with Tukey's post hoc test. ns, not significant (*p*>0.05); **p<0.05*; ***p*<0.01; ****p*<0.001; *****p*<0.0001. Abbreviations: WT, wild type; Vector, empty vector control; OE-PRKCB, PRKCB-overexpressing; LV-NC, non-targeting shRNA control; LV-PRKCB#1/2/3, PRKCB-targeting shRNA (sites #1, #2, #3).
