# Supplementary material for: FTO-mediated demethylation of PRKCB mRNA activates NF-κB to trigger alveolar fibrin deposition in ARDS
Source: Front Immunol. 2026 Oct 14;17:1960313. doi: 10.3389/fimmu.2026.1960313 (PMC13684130; doi:10.3389/fimmu.2026.1960313)
Supplement: Supplementary file 2 [file Table1.docx]

**a. Genes primer sequence**

| Genes | Forward Primer | Reverse Primer |
| --- | --- | --- |
| TF | ACTCAAGCACAGGAAAAAC | TGGAGAAAATCACGGCTTGTAC |
| PAI-1 | GCCTGTTCCACAAGTCTGATG | ATGAACATGCTGAGGGTTTTCG |
| METTL3 | CACTGCTTGGTTGGTGTCAA | CTCAATCTTTCGGGTGCCAG |
| METTL14 | GCGTCCTACTTCAGTGCCCC | TCCACCACCACGGTCAGACT |
| WTAP | GCAGCAACAGCAGGAATCTG | CTACACTCGGCTGCTGAACT |
| FTO | CGAGAGGCTGGCAGCATAC | CGGGGTGAGCACGTCTTTG |
| ALKBH5 | ACGGCCTCAGGACATCAAAG | AAGCATAGCTGGGTGGCAAT |
| GAPDH | AGATCCCTCCAAAATCAAGTGG | GGCAGAGATGATGACCCTTTT |
| PRKCB | ACTCAAGCACAGGAAAAAC | TGGAGAAAATCACGGCTTGTAC |
| Me- PRKCB | ACCAAAAGCTAGAGACAAGCGA | ACGAGAAGCCAGCAAATTCAT |

**b. Induction Scheme for FTO Overexpression**

ATGAAGCGCGTCCAGACCGCGGAGGAACGGGAGCGGGAAGCTAAGAAACTGAGGCTCCTCGAGGAGCTTGAAGACACTTGGCTTCCTTACCTGACCCCCAAAGATGACGAGTTCTATCAGCAGTGGCAGCTGAAATATCCTAAACTGGTTTTCCGAGAGGCTGGCAGCATACCCGAGGAGCTGCACAAAGAGGTCCCCGAGGCCTTTCTCACACTGCACAAGCATGGCTGCTTGTTTCGGGACCTGGTGAGGATCCAAGGCAAAGACGTGCTCACCCCGGTGTCTCGCATCCTCATTGGGGACCCCGGCTGCACCTACAAGTACTTGAACACCAGGCTCTTCACCGTGCCCTGGCCAGTGAAGGGCTGCACCATCAATTACACAGAGGCCGAGATTGCCGCCGCATGTCAGACCTTCCTCAAGCTCAATGACTACCTACAGGTCGAGACCATCCAGGCCTTGGAAGAACTGGCTATCAAAGAGAAGGCCAATGAAGACGCTGTGCCGTTGTGCATGGCAGAGTTCCCCAGGGCTGGCGTGGGACCGTCCTGCGATGATGAAGTGGACCTTAAGAGCAGAGCAGCCTACAACGTGACTTTGCTAAACTTCATGGATCCTCAGAAAATGCCGTACTTGAAAGAGGAGCCCTATTTCGGCATGGGGAAGATGGCGGTGAGCTGGCACCATGACGAGAACTTGGTGGACAGGTCAGCCGTGGCGGTGTACAGCTATAGCTGTGAAGGCTCCGAGGATGAAAGCGATGACGAGTCCAGCTTCGAAGGCAGAGATCCCGATACGTGGCATGTTGGTTTTAAGATCTCATGGGACATCGAGACGCCAGGCTTGACAATTCCTCTTCACCAGGGAGACTGCTATTTCATGCTGGATGACCTCAATGCCACCCACCAGCACTGTGTTTTGGCTGGCTCACAGCCTCGGTTTAGCTCCACCCACCGCGTGGCAGAGTGCTCAACAGGCACCTTGGATTATATCTTACAACGCTGCCAGTTGGCACTGCAGAATGTTCTCAATGACTCGGACAATGGCGACGTCTCGCTGAAGTCCTTCGAGCCTGCAGTTCTGAAACAAGGAGAAGAGATCCACAACGAGGTCGAGTTTGAGTGGCTGAGGCAGTTCTGGTTTCAAGGAAATCGATACAAAATTTGCACTGATTGGTGGTGTGAGCCCATGACTCAGCTGGAGGGGCTGTGGAAGAAGATGGAGAGTGTGACAAATGCCGTGCTTCGTGAAGTTAAGAGAGAGGGGCTCTCCGTGGAACAAAGGAGTGAAATTCTGTCTGCCGTCCTGATCCCACTCACCATGCGCCAGAATCTGAGGAAAGAATGGCACGCCAGGTGCCAGGCCCGAGTTGTCCGAACTCTACCAGCACAGCAGAAACCAGACTGCCGGCCATATTGGGAGAAGGATGACCCTTCTATGCCTCTGCCCTTTGACCTCACAGATGTGGTCTCTGAGATCAGAAGCCAGCTTCTGGAAGCAAGATCCTAG

**c. Knockdown of FTO**

| Genes | Interference sequence |
| --- | --- |
| FTO-shRNA1 | GCTGCACCTACAAGTACTTGA |
| FTO-shRNA2 | GCAGCCTACAACGTGACTTTG |
| FTO-shRNA1 | GCCTGCAGTTCTGAAACAAGG |
| FTO-shNC | GGTTCTCCGAACGTGTCACGT |

**d. Induction Scheme for PRKCB Overexpression**

GCCACCATGGCTGACCCGGCTGCGGGGCCGCCGCCGAGCGAGGGCGAGGAGAGCACGGTGCGCTTCGCCCGCAAAGGCGCCCTCCGGCAGAAGAACGTGCACGAGGTGAAGAACCACAAATTCACCGCCCGCTTCTTCAAGCAGCCCACCTTCTGCAGCCACTGCACCGACTTCATTTGGGGCTTAGGTTTGCAGGGATTCCAGAGTCAGGTCTGCTGCTTTGTTGTACACAAGCGCTGCCATGAATTCGTCACGTTCTCCTGCCCTGGTGCAGACAAGGGCCCGGCCTCTGATGACCCACGGAGCAAACACAAGTTTAAGATCCACACCTACTCCAGCCCTACCTTCTGTGACCACTGTGGATCACTGCTGTATGGGCTCATCCACCAGGGGATGAAATGCGACACCTGTATGATGAATGTCCACAAGCGCTGCGTGATGAACGTCCCCAGCCTCTGTGGCACCGACCACACAGAACGCCGTGGCCGCATCTACATCCAGGCCCACATCGACAGGGAGGTCCTCATCGTTGTTGTAAGAGATGCTAAAAATCTGGTACCTATGGACCCCAACGGCTTGTCAGATCCCTACGTAAAACTGAAACTGATCCCTGATCCCAAAAGTGAGAGCAAGCAGAAGACCAAGACTATCAAATGCTCCCTCAACCCGGAGTGGAACGAAACCTTCAGATTTCAGCTGAAGGAATCAGACAAAGACAGAAGACTGTCCGTAGAGATCTGGGATTGGGACCTGACCAGCAGGAATGACTTCATGGGATCTCTGTCGTTTGGGATTTCAGAACTACAGAAAGCCGGAGTGGATGGCTGGTTCAAGTTACTAAGCCAGGAAGAAGGCGAGTACTTTAATGTGCCGGTGCCGCCGGAAGGAAGCGAGGGCAATGAAGAGCTGCGGCAGAAGTTTGAGAGAGCCAAGATTGGCCAAGGTACCAAGGCTCCAGAAGAAAAGACAGCGAACACTATATCCAAATTTGACAACAATGGCAACAGGGACCGGATGAAACTGACCGATTTTAACTTCCTGATGGTGCTGGGGAAAGGCAGCTTTGGCAAGGTCATGCTCTCAGAGCGGAAGGGTACAGATGAACTCTATGCCGTGAAGATCCTGAAGAAAGATGTGGTGATCCAAGATGACGATGTGGAGTGCACAATGGTGGAGAAGAGGGTGCTGGCCCTGCCTGGGAAGCCCCCATTCCTGACTCAGCTCCATTCCTGCTTCCAGACCATGGACCGCCTCTACTTTGTGATGGAGTATGTGAACGGGGGCGACCTCATGTACCACATCCAACAAGTTGGCCGTTTCAAGGAGCCCCATGCTGTATTTTACGCTGCAGAGATTGCCATCGGTCTTTTCTTCTTGCAGAGCAAGGGCATCATTTACCGTGACCTGAAACTTGACAACGTGATGCTGGATTCCGAGGGGCACATCAAAATCGCTGACTTTGGCATGTGTAAAGAGAATATCTGGGATGGGGTGACAACCAAGACATTCTGTGGCACTCCAGACTACATTGCCCCAGAGATCATTGCTTATCAGCCCTACGGGAAGTCTGTGGACTGGTGGGCGTTTGGAGTCCTGCTGTATGAAATGTTGGCTGGCCAGGCACCTTTTGAAGGGGAGGATGAGGATGAACTCTTCCAGTCAATCATGGAGCACAACGTGGCGTATCCCAAGTCCATGTCTAAGGAAGCTGTGGCAATCTGCAAAGGGCTAATGACCAAACACCCAGGCAAGCGCCTGGGTTGTGGGCCTGAAGGGGAACGAGACATTAAGGAGCATGCATTTTTCCGGTATATCGACTGGGAGAAACTCGAACGCAAGGAGATTCAGCCACCTTATAAACCAAAAGCTAGAGACAAGCGAGACACCTCCAACTTCGACAAAGAGTTCACCAGGCAGCCTGTGGAACTGACTCCCACTGACAAACTCTTCATCATGAACTTGGACCAAAATGAATTTGCTGGCTTCTCGTATACTAACCCAGAGTTTGTCATTAATGTGTAG

**e. Knockdown of PRKCB**

| Genes | Interference sequence |
| --- | --- |
| PRKCB-shRNA1 | GCCACTGCACCGACTTCATTT |
| PRKCB-shRNA2 | GCTTAGGTTTGCAGGGATTCC |
| PRKCB-shRNA1 | GCCATGAATTCGTCACGTTCT |
| PRKCB-shNC | GGTTCTCCGAACGTGTCACGT |

**f. PRKCB m6A modification site**

ATGGCTGACCCGGCTGCGGGGCCGCCGCCGAGCGAGGGCGAGGAGAGCACGGTGCGCTTCGCCCGCAAAGGCGCCCTCCGGCAGAAGAACGTGCACGAGGTGAAGAACCACAAATTCACCGCCCGCTTCTTCAAGCAGCCCACCTTCTGCAGCCACTGCACCGACTTCATTTGGTTAGGTTTGCAGGGATTCCAGAGTGCAGTCTGCTGCTTTGTTGTACACAAGCGCTGCCATGAATTCGTCACGTTCTCCTGCCCTGGTGCAGACAAGGGCCCGGCCTCTGATGACCCACGGAGCAAACACAAGTTTAAGATCCACACCTACTCCAGCCCTACCTTCTGTGACCACTGTGGATCACTGCTGTATGGGCTCATCCACCAGGGGATGAAATGCGACACCTGTATGATGAATGTCCACAAGCGCTGCGTGATGAACGTCCCCAGCCTCTGTGGCACCGACCACACAGAACGCCGTGGCCGCATCTACATCCAGGCCCACATCGACAGGGAGGTCCTCATCGTTGTTGTAAGAGATGCTAAAAATCTGGTACCTATGGACCCCAACGGCTTGTCAGATCCCTACGTAAAACTGAAACTGATCCCTGATCCCAAAAGTGAGAGCAAGCAGAAGACCAAGACTATCAAATGCTCCCTCAACCCGGAGTGGAACGAAACCTTCAGATTTCAGCTGAAGGAATCAGACAAAGACAGAAGACTGTCCGTAGAGATCTGGGATTGGGACCTGACCAGCAGGAATGACTTCATGGGATCTCTGTCGTTTGGGATTTCAGAACTACAGAAAGCCGGAGTGGATGGCTGGTTCAAGTTACTAAGCCAGGAAGAAGGCGAGTACTTTAATGTGCCGGTGCCGCCGGAAGGAAGCGAGGGCAATGAAGAGCTGCGGCAGAAGTTTGAGAGAGCCAAGATTGGCCAAGGTACCAAGGCTCCAGAAGAAAAGACAGCGAACACTATATCCAAATTTGACAACAATGGCAACAGGGACCGGATGAAACTGACCGATTTTAACTTCCTGATGGTGCTGGGGAAAGGCAGCTTTGGCAAGGTCATGCTCTCAGAGCGGAAGGGTACAGATGAACTCTATGCCGTGAAGATCCTGAAGAAAGATGTGGTGATCCAAGATGACGATGTGGAGTGCACAATGGTGGAGAAGAGGGTGCTGGCCCTGCCTGGGAAGCCCCCATTCCTGACTCAGCTCCATTCCTGCTTCCAGACCATGGACCGCCTCTACTTTGTGATGGAGTATGTGAACGGGGGCGACCTCATGTACCACATCCAACAAGTTGGCCGTTTCAAGGAGCCCCATGCTGTATTTTACGCTGCAGAGATTGCCATCGGTCTTTTCTTCTTGCAGAGCAAGGGCATCATTTACCGTGACCTGAAACTTGACAACGTGATGCTGGATTCCGAGGGGCACATCAAAATCGCTGACTTTGGCATGTGTAAAGAGAATATCTGGGATGGGGTGACAACCAAGACATTCTGTGGCACTCCAGACTACATTGCCCCAGAGATCATTGCTTATCAGCCCTACGGGAAGTCTGTGGACTGGTGGGCGTTTGGAGTCCTGCTGTATGAAATGTTGGCTGGCCAGGCACCTTTTGAAGGGGAGGATGAGGATGAACTCTTCCAGTCAATCATGGAGCACAACGTGGCGTATCCCAAGTCCATGTCTAAGGAAGCTGTGGCAATCTGCAAAGGGCTAATGACCAAACACCCAGGCAAGCGCCTGGGTTGTGGGCCTGAAGGGGAACGAGACATTAAGGAGCATGCATTTTTCCGGTATATCGACTGGGAGAAACTCGAACGCAAGGAGATTCAGCCACCTTATAAACCAAAAGCTAG**AGACA**AGCG**AGACA**CCTCCAACTTCGACAAAGAGTTCACCAGGCAGCCTGTG**GAACT**GACTCCCACTGAC**AAACT**CTTCATCAT**GAACT**T**GGACC**AAAATGAATTTGCTGGCTTCTCGTATACTAACCCAGAGTTTGTCATTAATGTG
