# Supplementary material for: FTO-mediated demethylation of PRKCB mRNA activates NF-κB to trigger alveolar fibrin deposition in ARDS
Source: Front Immunol. 2026 Oct 14;17:1960313. doi: 10.3389/fimmu.2026.1960313 (PMC13684130; doi:10.3389/fimmu.2026.1960313)
Supplement: Supplementary file 3 [file Table2.docx]

**Antibody information**

| Antibody name | Dilution Ratio | Production company |
| --- | --- | --- |
| FTO Antibody | 1:500 | Abcam Company, USA |
| NF-κB p-IKKβ Antibody | 1:1000 | Abcam Company, USA |
| NF-κB IKKβ Antibody | 1:3000 | Wuhan Sanying Biotechnology Co., LTD |
| NF-κB p-p65 Antibody | 1:1000 | Cell Signaling Technology of the United States |
| NF-κB P65 Antibody | 1:1000 | Wuhan Sanying Biotechnology Co., LTD |
| TF Antibody | 1:3000 | NOVUS Corporation of the United States |
| PAI-1 Antibody | 1:600 | Wuhan Sanying Biotechnology Co., LTD |
| GAPDH Antibody | 1:5000 | Abcam Company, USA |
| Collagen Ⅲ Antibody | 1:100 | Abcam Company, USA |
| PRKCB Antibody | 1:1000 | Abcam Company, USA |
| Sheep anti-rabbit secondary antibody | 1:10000 | Jackson Company of the United States |
| Sheep anti-mouse secondary antibody | 1:10000 | Jackson Company of the United States |
