# Supplementary material for: FTO-mediated demethylation of PRKCB mRNA activates NF-κB to trigger alveolar fibrin deposition in ARDS
Source: Front Immunol. 2026 Oct 14;17:1960313. doi: 10.3389/fimmu.2026.1960313 (PMC13684130; doi:10.3389/fimmu.2026.1960313)
Supplement: Supplementary file 5 [file Table4.docx]

Table 1 Lung injure scoring system

| Parameter | Score per field | | |
| --- | --- | --- | --- |
|  | 0 | 1 | 2 |
| A. Neutrophils in the alveolar space | none | 1-5 | ＞5 |
| B. Neutrophils in the interstitial space | none | 1-5 | ＞5 |
| C. Hyaline membranes | none | 1 | ＞1 |
| D. Proteinaceous debris filling the airspaces | none | 1 | ＞1 |
| E. Alveolar septal thickening | ＜2x | 2x-4x | ＞4x |

Score = [(20 × A) + (14 × B) + (7 × C) + (7 × D) + (2 × E)] / (number of fields × 100)
